# Artificial intelligence based multispecialty mortality prediction models for septic shock in a multicenter retrospective study

**DOI:** 10.1038/s41746-025-01643-w

**Published:** 2025-04-28

**Authors:** Shurui Wang, Xinyi Liu, Shaohua Yuan, Yi Bian, Hong Wu, Qing Ye

**Affiliations:** 1 Tongji Hospital, Tongji Medical College, Huazhong University of Science and Technology, Wuhan, China; 2 School of Public Health, Tongji Medical College, Huazhong University of Science and Technology, Wuhan, China; 3 School of Cyber Science and Engineering, Zhengzhou University, Zhengzhou, China; 4 Department of Critical Care Medicine, Tongji Hospital, Tongji Medical College, Huazhong University of Science and Technology, Wuhan, Hubei China; 5 Department of Emergency Medicine, Tongji Hospital, Tongji Medical College, Huazhong University of Science and Technology, Wuhan, Hubei China; 6 School of Medicine and Health Management, Tongji Medical College, Huazhong University of Science and Technology, Wuhan, China

**Keywords:** Machine learning, Diseases, Risk factors

## Abstract

Septic shock is one of the most lethal conditions in ICU, and early risk prediction may help reduce mortality. We developed a TOPSIS-based Classification Fusion (TCF) model to predict mortality risk in septic shock patients using data from 4872 ICU patients from February 2003 to November 2023 across three hospitals. The model integrates seven machine learning models via the Technique for Order Preference by Similarity to an Ideal Solution (TOPSIS), achieving AUCs of 0.733 in internal validation, 0.808 in the pediatric ICU, 0.662 in the respiratory ICU, with external validation AUCs of 0.784 and 0.786, respectively. It demonstrated high stability and accuracy in cross-specialty and multi-center validation. This interpretable model provides clinicians with a reliable early-warning tool for septic shock mortality risk, facilitating early intervention to reduce mortality.

## Introduction

Septic shock is a severe syndrome of circulatory and cellular metabolic disorders caused by sepsis, also known as infectious shock, a type of distributed shock^1^. The clinical diagnostic criteria for septic shock in the third edition of the International Consensus on the Definition of Sepsis and Infectious Shock, published in 2016, are persistent hypotension despite adequate volume resuscitation in patients with sepsis, the need for vasoconstrictor medication to maintain a mean arterial pressure of not less than 65 mmHg and a serum lactate level greater than 2 mmol/L^2^.

Septic shock, which is characterized by high mortality and difficulty in early warning, has always received considerable attention from researchers. A retrospective cohort study using health record data reported an in-hospital mortality rate of 15% in patients with septic shock^3^; another retrospective study using the National Intensive Care Database reported a mortality rate of 55.5% in septic shock patients^4^. In Michael Bauer’s review of studies, the 30-day observed mortality rate in patients with septic shock was 34.73% (32.61–36.85%)^5^. Early warning of patient outcomes in septic shock can give physicians more time to intervene and carry out more aggressive therapeutic measures, thereby reducing mortality^6,7^. However, due to the complexity of the disease in septic shock patients, there is currently no effective model to predict the prognosis of septic shock patients. If the disease progression of septic shock patients can be predicted in advance, then clinicians can closely monitor patients at higher risk of death earlier and take more aggressive treatment measures.

Septic shock, a subtype of sepsis with severe circulatory and cellular metabolic abnormalities and an unstable hemodynamic state, has become the leading cause of death in ICU patients. Early warning and intervention in sepsis progression have the potential to reduce mortality, so sepsis-related early warning efforts have gained widespread attention from researchers. Several scholars have already used clinical data to construct artificial intelligence models to predict sepsis^8–10^. These studies typically use patient vital sign data, laboratory test data, and various types of therapeutic data from electronic medical records to build machine learning models^8,11^. Some of these studies achieved early warning of sepsis by mining publicly available databases or retrospective cohorts to develop machine learning models^11,12^. These studies achieved prediction of disease progression in patients with sepsis by constructing advanced machine learning models, which brought time for physicians to intervene clinically and reduced mortality^13^. However, most of these studies have only been conducted on septic patients and are not applicable to septic shock patients. In addition, many of these studies have not been applied in clinical practice due to the small sample size, the use of a single machine learning model and the lack of extensive multicenter validation.

In order to overcome the problem of poor performance of small cohort and single classification models in clinical scenarios, we aim to construct a robust fusion model with high generalization ability based on several base classification models through an efficient fusion strategy. This model is used to accurately predict the 28-day risk of death in ICU septic shock patients. In addition, we will transparently visualize the inference process of our model through feature importance visualization to enhance the credibility of our model and provide ICU physicians with an efficient, stable, and trustworthy clinical decision-making tool.

## Results

### Study population characteristics and cohort composition

**Fig. 1:**
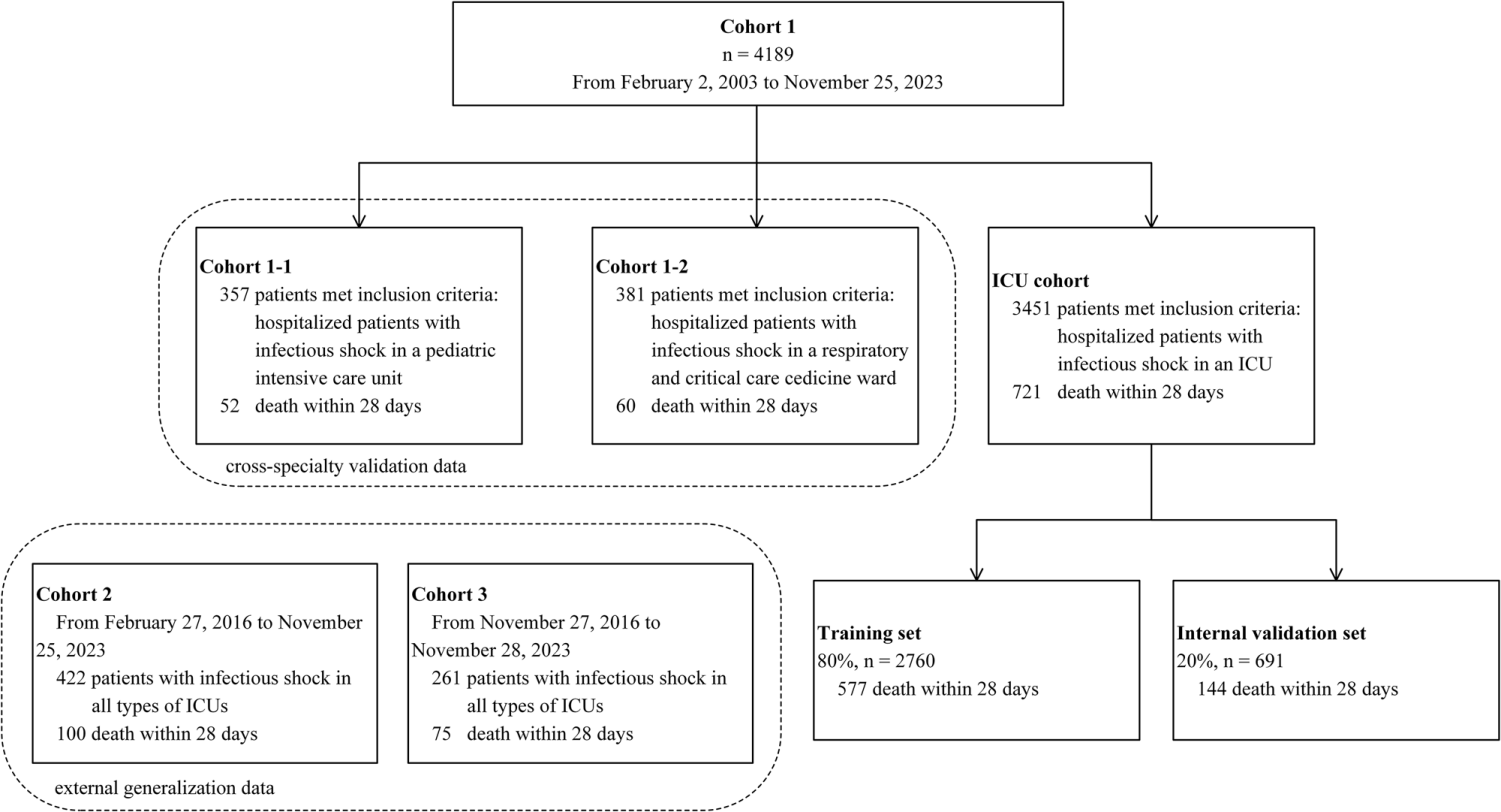
Study cohort selection and dataset partitioning flowchart. Data from three central cohorts (Cohort 1, Cohort 2, and Cohort 3) were included in the study. Cohorts 2 and 3 were used to assess the model’s generalizability across different centers. Subsets from Cohort 1 (Cohort 1-1 and Cohort 1-2) were extracted to further evaluate its applicability in different specialized intensive care units (ICUs). Model development data also came from Cohort 1, which included patients from general ICUs. This dataset was split into 70% training data and 30% internal validation data. The training data were used to build the base model, while the internal validation data tested the model’s effectiveness and demonstrated its performance in real-world scenarios.

The ICU cohort consisted of 3451 participants, including 721 outcome-positive participants and 2730 outcome-negative participants. The pediatric intensive care unit cohort (cohort 1-1) comprised 357 individuals with 52 positive results (14.57%). The respiratory intensive care unit cohort (cohort 1-2) comprised 381 individuals with 60 positive results (15.75%). The first external validation cohort, cohort 2, included 422 participants, with 100 outcome-positive participants and 322 outcome-negative participants. The second external validation cohort, cohort 3, included 261 participants, consisting of 75 outcome-positive participants and 186 outcome-negative participants (Fig. 1). The advantage of our data is that the data related to model construction (Cohort 1) comes from general ICU, featuring a complex and diverse participant background, which can theoretically contribute to constructing a widely applicable septic shock prediction model. In addition to multicenter validation, we also conducted cross-specialty validation, further demonstrating the wide applicability of the model. The basic profile of the study population is shown in Table 1, and the results of the hypothesis tests for each group of features are shown in (Supplementary Table 1).

**Table 1:** Baseline characteristics of included population

	Cohort 1				External validation set	
	Training set (*n* = 2760)	Internal validation set (*n* = 691)	Cohort 1-1 (*n* = 357)	Cohort 1-2 (*n* = 381)	Cohort 2 (*n* = 422)	cohort 3 (*n* = 261)
Age, years	59.5 (48.0–69.0)	59.0 (47.5–68.0)	3.0 (0.0–7.0)	65.0 (51.0–73.0)	64.5 (52.3–75.0)	65.0 (55.0–73.0)
Gender						
Female	1084 (39.28%)	293 (42.40%)	128 (35.85%)	124 (32.55%)	172 (40.76%)	102 (39.08%)
Male	1676 (60.72%)	398 (57.60%)	229 (64.15%)	257 (67.45%)	250 (59.24%)	159 (60.92%)
LOS, h	256.33 (91.58–497.88)	230.69 (68.43–456.38)	357.50 (209.05–635.47)	325.70 (139.97–576.00)	190.46 (79.37–349.22)	135.03 (47.47–278.70)
hs-CRP, mg/L	130.48 (66.70–199.08)	123.60 (59.10–198.15)	66.74 (22.30–127.50)	108.10 (55.30–154.90)	115.02 (52.78–192.38)	130.60 (57.50–195.60)
ALT, IU/L	30 (15.00–88.09)	29 (15–72)	29 (15–81)	27 (14–57)	25 (15–55)	29 (15–78)
Na, mmol/L	134.45 (1.39–141.10)	134.20 (1.37–140.70)	132.20 (103.68–136.30)	134.50 (1.41–139.40)	132.25 (1.37–138.50)	137.80 (133.90–141.60)
Lac, mmol/L	3.38 (1.97–5.62)	3.07 (1.80–5.41)	3.81 (2.48–5.05)	2.79 (1.32–5.01)	2.66 (1.53–4.41)	2.89 (1.70–4.91)
PLR	192.41 (85.34–362.04)	178.87 (75.08–336.56)	116.67 (62.50–205.68)	213.75 (125.64–339.87)	207.86 (102.21–368.33)	194.66 (111.11–352.78)
LMR	1.57 (0.86–3.06)	1.58 (0.83–3.20)	4.20 (1.98–1.97)	1.52 (0.94–2.58)	1.31 (0.75–2.60)	1.32 (0.74–2.64)
Ventilation time, h						
Ventilator Recipients	1512 (54.78%)	399 (57.74%)	116 (32.49%)	158 (41.47%)	162 (38.39%)	123 (47.13%)
	62 (18–154)	62 (15–146)	82 (18–197.13)	157.50 (58.75–372.00)	71 (24.5–211.5)	58 (17–135)
Non-Ventilator Recipients	1248 (45.22%)	292 (42.56%)	241 (67.51%)	223 (58.53%)	260 (61.61%)	138 (52.87%)
Oxygen flow rate via nasal cannula, L/min						
Nasal Cannula Recipients	602, 21.81%	132, 19.10%	67, 18.77%	37, 9.71%	93, 22.04%	61, 23.37%
	3 (3–5)	3 (3–5)	1 (0.5–1)	3 (2–5)	2 (2–3)	3 (2–5)
Non-Nasal Cannula Recipients	2158, 78.19%	559, 80.90%	290, 81.23%	344, 90.29%	329, 77.96%	200, 76.63%
Frequency of hemodialysis						
Hemodialysis Recipients	1217, 44.09%	317, 45.88%	18, 5.04%	64, 16.79%	154, 36.49%	138, 52.87%
	2 (1–3)	2 (1–3)	3.5 (2–7.5)	3.50 (2–8)	2 (1–3)	2 (1–3)
Non-Hemodialysis Recipients	1543, 55.91%	374, 54.12%	339, 94.96%	317, 83.20%	268, 63.51%	123, 47.13%
Clinical Outcomes						
Death Within 28 Days	577 (20.91%)	144 (20.84%)	52 (14.57%)	60 (15.75%)	100 (23.70%)	75 (28.74%)
Others	2183 (79.09%)	547 (79.16%)	305 (85.43%)	321 (84.25%)	322 (76.30%)	186 (71.26%)

Data presented as median (IQR) or *n* (%). LOS = length of stay. hs-CRP = high-sensitivity C-reactive protein. ALT = alanine aminotransferase. Na = sodium ion. Lac = lactate. PLR = platelet-to-lymphocyte ratio. LMR = lymphocyte-to-monocyte ratio.

### Feature selection and optimization process

**Fig. 2:**
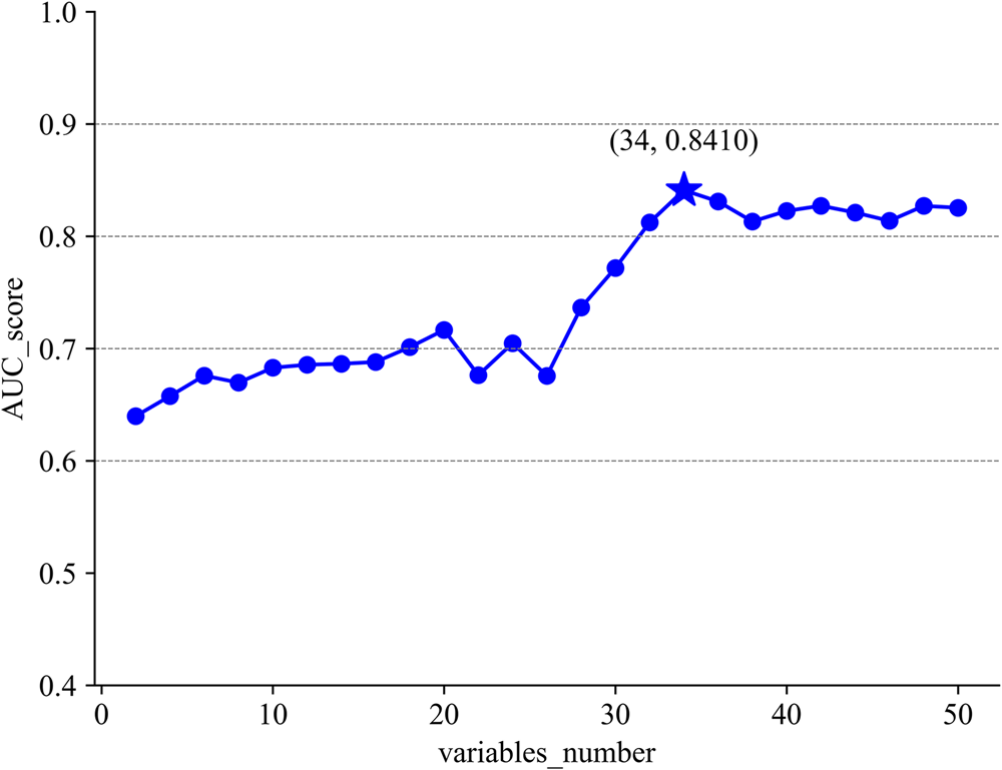
Stepwise feature selection performance. Features were ranked based on information entropy, with more valuable features prioritized and sequentially incorporated into the model. When the number of variables exceeded 34, the AUC gain no longer increased.

Sequence characterization was performed on the training data (Supplementary Fig. 1), removing 23 features with a missing rate higher than 30%, leaving 70 features. The variance was calculated for 23 of the Boolean features, leaving 61 features after screening. The correlation coefficients were calculated (Supplementary Fig. 2), and there were 12 groups of features that needed to be filtered in pairs according to the principle of higher AUC (Supplementary Tables 2 and 3), at which point there were 50 features left. The results of the four algorithms for imputing missing values are shown in Supplementary Table 4, and the prediction effect of the Multiple Imputation method based on Logistic Regression (AUC = 0.844) is significantly higher than that of the other methods. Finally, the entropy values of the remaining features were calculated and modeled in order from largest to smallest. Figure 2 shows how the model effect varies with the increase of features. When the number of features is 34, the AUC reaches the optimal, and these features are the final input features of seven sub-models and TCF. The 34 indicators are: Age, Surgical history (SH), Temperature (Temp), Diastolic blood pressure (DBP), Length of stay in the ICU until discharge (LOC-ICU), Oxygen uptake (litres/minute) (OU), Duration of mechanical ventilation (DOMV), High-sensitivity C-reactive protein (hs-CRP), Fibrinogen (FBG), Prothrombin time (PT), Activated partial thromboplastin time (APTT), Alanine aminotransferase (ALT), Direct bilirubin (DBIL), Creatinine (Cr), Uric acid (UA), Total calcium (Tca), Potassium ion (K), Sodium ion (Na), Lactate (Lac), Albumin (ALB), Glucose (Glu), Lymphocyte count (LYM#), White blood cell count (WBC#), Haemoglobin (Hb), Total cholesterol (TC), High-density lipoprotein cholesterol (HDL-C), Monocyte count (MONO), Procalcitonin (PCT), Number of resuscitations (NOR), Number of blood purifications (NOBP), NLR, PLR, LMR, PAR (Supplementary Table 5).

### TCF model performance and sub-model comparisons

Parameter optimization and sub-model training were performed using the training set (Supplementary Tables 6 and 7). Subsequently, model fusion and weight assignment were conducted based on the internal validation set (Supplementary Table 8). The fusion algorithm for the TCF model is as follows:

1 $$\begin{array}{c}{p}_{j}={p}_{{DT}}\times 0.141+{p}_{{RF}}\times 0.153+{p}_{{XGB}}\times 0.155+{p}_{{LGMB}}\times 0.151\\ +{p}_{{SVM}}\times 0.089+{p}_{{NB}}\times 0.153+{p}_{{GBDT}}\times 0.158\end{array}$$

where $j$ represents the *j-th* predicted sample, and p represents the probability of predicting a positive result.

**Fig. 3:**
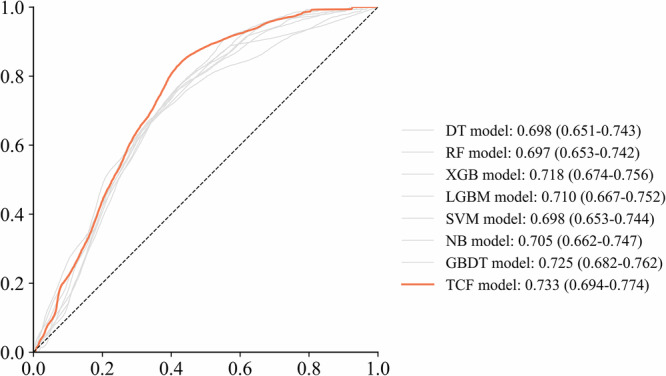
Comparison of ROC curves between TCF and its sub-models. A comparison of ROC curves between the TCF model and sub-models in the internal validation set demonstrated superior performance of the TCF model, as evidenced by both the curve trajectories and AUC values.

The model’s performance was validated as shown in Table 2. TCF outperforms the sub-models in terms of two comprehensive evaluation indices: AUC 0.733 (95% CI: 0.694–0.774) and F1-score 0.458 (0.398–0.520). Additionally, ACC 0.686 (0.654–0.721) and PRE 0.358 (0.302–0.419) are also higher than most sub-models, showcasing excellent classification capabilities. LGBM demonstrates the best positive identification ability (SEN 0.735 (0.664-0.808)), while SVM shows the best negative identification ability (SPE 0.907 (0.882–0.931)). However, models that excel in certain indices may lack in others. For instance, SVM has a weak positive identification ability (SEN 0.236 (0.171–0.306)), and LGBM performs worse than TCF in terms of the comprehensive indices AUC, F1-score, and ACC. The comparison of ROC curves between the TCF model and sub-models is shown in Fig. 3. The calibration curve and Decision Curve Analysis (DCA) can be found on Supplementary Figs. 3 and 4, respectively. Although the TCF model does not achieve optimal performance in identifying positives (SEN 0.640 (0.557–0.712)) and negatives (SPE 0.700 (0.663–0.736)), it can integrate the biased identification effects of sub-models, resulting in the best overall performance. The calibration curve and DCA also demonstrate that the predictions of the TCF model are consistent with the actual outcomes, indicating its potential clinical utility within a certain range. It can assist decision-makers in making more beneficial decisions.

**Table 2:** Performance of sub-models and TCF on the internal validation set

Models	AUC	F1-score	ACC	PRE	SEN	SPE
DT	0.698 (0.651, 0.743)	0.426 (0.367, 0.484)	0.674 (0.638, 0.708)	0.336 (0.278, 0.390)	0.585 (0.503, 0.660)	0.698 (0.657, 0.734)
RF	0.697 (0.653, 0.742)	0.447 (0.391, 0.504)	0.667 (0.634, 0.703)	0.344 (0.291, 0.399)	0.646 (0.569, 0.725)	0.675 (0.637, 0.712)
XGB	0.718 (0.674, 0.756)	0.456 (0.396, 0.511)	0.654 (0.619, 0.689)	0.339 (0.284, 0.392)	0.701 (0.624, 0.770)	0.642 (0.600, 0.682)
LGBM	0.710 (0.667, 0.752)	0.455 (0.394, 0.511)	0.632 (0.596, 0.669)	0.330 (0.276, 0.380)	0.735 (0.664, 0.808)	0.605 (0.562, 0.646)
SVM	0.698 (0.653, 0.744)	0.296 (0.224, 0.374)	0.767 (0.737, 0.796)	0.402 (0.303, 0.507)	0.236 (0.171, 0.306)	0.907 (0.882, 0.931)
NB	0.705 (0.662, 0.747)	0.456 (0.397, 0.514)	0.645 (0.609, 0.680)	0.336 (0.282, 0.388)	0.714 (0.640, 0.795)	0.627 (0.589, 0.668)
GBDT	0.725 (0.682, 0.762)	0.453 (0.391, 0.512)	0.687 (0.651, 0.722)	0.355 (0.297, 0.415)	0.623 (0.544, 0.704)	0.703 (0.667, 0.743)
TCF	0.733 (0.694, 0.774)	0.458 (0.398, 0.520)	0.686 (0.654, 0.721)	0.358 (0.302, 0.419)	0.640 (0.557, 0.712)	0.700 (0.663, 0.736)

Performance metrics (Area under ROC curve (AUC), F1-score, Precision (PRE), Accuracy (ACC), Sensitivity (SEN), and Specificity (SPE)) with 95% CIs were computed via 1000 bootstrap resamplings for the internal validation set across seven models, including TCF model. The TCF model achieved superior performance.

### Feature importance and model interpretation

**Fig. 4:**
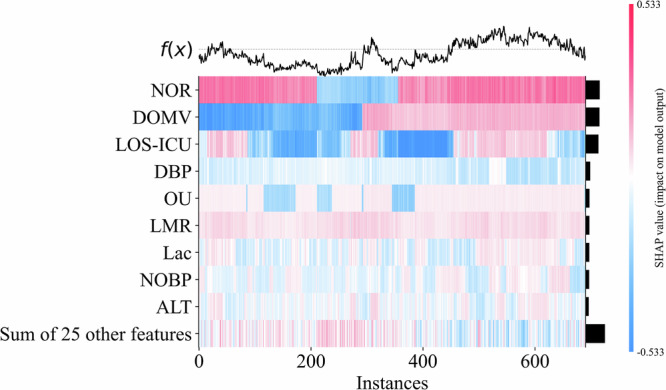
Optimal sub-model (GBDT) SHAP feature importance heatmap. The GBDT model demonstrated the best performance among all sub-models. Feature importance was visualized using SHapley Additive exPlanation (SHAP) summary plots, revealing that the number of resuscitations (NOR), duration of mechanical ventilation (DOMV), length of stay in the ICU until discharge (LOC-ICU), diastolic blood pressure (DBP), along with other clinical features, had the most significant impact on outcomes in the GBDT model.

Since the TCF models are fusion models, their feature importance consists of all sub-model features and their weights. In order to understand the inference process of our models more deeply, we chose the GBDT model, which has the best performance in terms of AUC, and visualized its feature importance by drawing a SHAP feature importance heatmap, as shown in Fig. 4 (Supplementary Fig. 5). The results showed that in the GBDT model, Number of resuscitations (NOR), Duration of mechanical ventilation (DOMV), Length of stay in the ICU until discharge (LOC-ICU), Diastolic blood pressure (DBP) and other clinical characteristics have a greater impact on outcome. The weighted feature importance of the TCF model is on Supplementary Table 9, which is essentially consistent with GBDT, and the feature importance ranking of the ensemble model also has reference value.

### Multicenter validation and generalizability

**Fig. 5:**
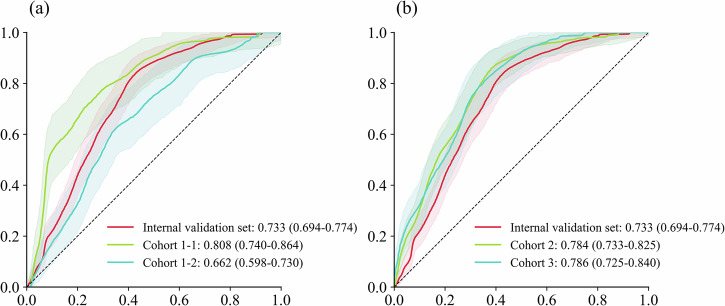
Comparison of ROC curves for multicenter data. **a** The difference in the ability of cross-specialty data and the internal validation set to distinguish between positive and negative patients. **b** The difference in the ability of multicenter data and the internal validation set to distinguish between positive and negative patients. ROC curves were used for visualization, with the shaded areas representing the 95% confidence intervals.

**Fig. 6:**
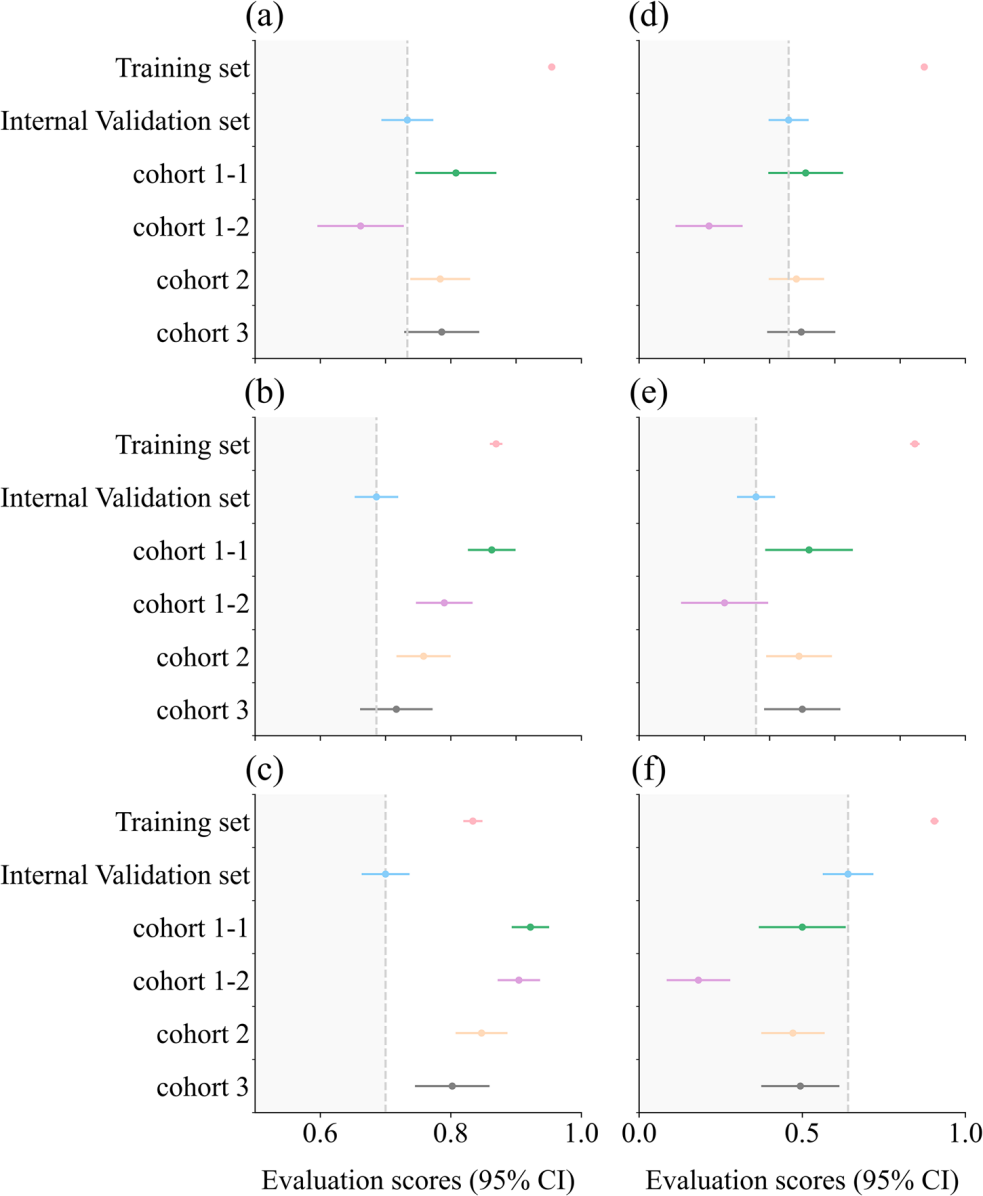
Performance of six indices (95% CI) across all datasets. The performance (95% CI) of the six indices across all datasets. **a** shows the boxplot of the Area Under the Curve (AUC), (**b**) shows the boxplot of Accuracy (ACC), (**c**) shows the boxplot of Specificity (SPE), (**d**) shows the boxplot of the F1-score, (**e**) shows the boxplot of Precision (PRE), and (**f**) shows the boxplot of Sensitivity (SEN). The gray dashed line represents the results of the internal validation set. Evaluation scores for the other datasets fall within the dark gray area, indicating a decline in performance compared to the internal validation set.

The results of multicenter validation can more accurately demonstrate the predictive performance of TCF model, and there is heterogeneity among different datasets. In most studies, the predictive effect on multicenter data is slightly inferior to that on the training set and internal validation set. In this study, compared to the internal validation set, the AUC slightly decreased on cohort 1-2 (AUC 0.662 (95% CI: 0.598–0.730)) (Fig. 5a), while the AUC showed significant improvement on cohort 1-1 (AUC 0.808 (0.740–0.864)), cohort 2 (AUC 0.784 (0.733–0.825)), and cohort 3 (AUC 0.786 (0.725–0.840)) (Fig. 5). Figure 6 and Supplementary Table 10 further demonstrate the comparison of results on six indices across different datasets. Due to the limited sample size of multicenter data and fewer positive samples, SEN decreases, but SPE significantly improves, with recognition capability reaching over 80%. Therefore, we specifically combined the four external validation datasets for prediction. The sample size at this point was 1421 (including 287 positive events), with an AUC of 0.7705 (0.7417–0.7983). We also plotted the calibration curve (Supplementary Fig. 6). This indicates that the TCF model can effectively distinguish septic shock patients with low-risk factors and possesses excellent calibration capability. We also performed DCA on the combined dataset (Supplementary Fig. 7). The net benefit of the model intervention was higher than the “treat-all” and “treat-none” strategies across most threshold probability ranges. Moreover, within the threshold probability range (~0.1–0.5), the model demonstrated even greater net benefit. This indicates that the predictions of the TCF model have significant clinical value for decision-making within this range. Most multicenter data perform better than the internal validation set in terms of AUC, F1-score, ACC, PRE, and SPE. Although cohort 1-2 shows a slight decrease in AUC and F1-score, it still achieves good classification capability. Overall, TCF has an unexpectedly strong generalization ability, which is also attributed to the complex patient background in the ICU cohort.

## Discussion

In this study, we developed the TCF model for accurately predicting the 28-day risk of death for septic shock patients based on 34 features. Adopting TOPSIS to fuse the seven base models, TCF models achieved consistent and good performance on both the internal set and the two external validation sets, outperforming all individual models before fusion in predicting 28-day risk of death for septic shock patients.

The risk of death in patients with septic shock is difficult to differentiate due to the severity, complexity, and overall high mortality of sepsis^14,15^. Early recognition is also challenging because sepsis shares similar clinical signs (e.g., changes in vital signs), symptoms (e.g., fever), and molecular manifestations (e.g., host response dysregulation^16^. The strength of the TCF model lies in the fact that it provides clinicians in ICU with a tool to assist them in real-time prediction of a patient’s risk of death over a period of 28 days, especially in the deteriorating early stages of disease progression or in primary care settings with limited clinical experience. Interpretable work on our model features has made an outstanding contribution to predicting the risk of death in septic shock and has helped clinicians better understand the mechanisms of sepsis and septic shock progression.

Our TCF model showed strong stability and generalization ability. We have not only validated our TCF model across specialties but also across multiple centers. On the internal validation set, the AUC of the TCF model was 0.733 (95% CI 0.694–0.774). On the pediatric and respiratory ICU datasets, the AUC was 0.808 (0.740–0.864) and 0.662 (0.598–0.730), respectively. In both external validation sets, the AUC similarly achieved 0.784 (0.733–0.825) and 0.786 (0.725–0.840). The results show that the model exhibits excellent performance on different ICU patient populations. Although the multicenter data can validate the stability of the model, it can likewise present us with several problems that are not friendly to building AI models. Our data issues included an imbalance in the proportion of positives among septic shock patients, a large variation in sample size, and many missing values (Supplementary Table 11). In order to address these data issues and ensure model robustness, extensive and varied data cleaning was performed. The most appropriate data preprocessing method was selected by comparing the results of different cleaning methods on the study data.

Our TCF model exhibits high prediction accuracy, which is inextricably linked to the model fusion strategy we adopted. In this study, we used the TOPSIS method to fuse seven base models to construct the TCF model. The model brings together the strengths of each base model and obtains the best prediction by weighting the contributions of each model. The TCF model provides a more reliable and consistent prediction of the risk of septic shock death for multicenter data than known base prediction models (Supplementary Table 7).

Artificial intelligence predictive models based on electronic medical record data have been employed in many diseases, including several studies on the prediction of the risk of death from septic shock^17,18^. Zheng et al. developed six machine-learning models that predicted 28-day mortality in patients with septic shock across 46 features, including markers of inflammation, organ dysfunction, or injury^19^. The effect of a certain drug intervention on septic shock has also been studied using Artificial Neural Network^20^. However, these existing studies have not been clinically applied due to problems such as small cohort size and lack of extensive multicenter validation. In contrast, the TCF model constructed in this study was externally validated not only by two cohorts but also by two different specialties. Therefore, our model has higher credibility and generalizability.

Feature importance ranking not only enhances the transparency and credibility of clinical predictive models but also provides valuable insight and support for medical practice. In this study, we performed feature importance ranking on the TCF model to enhance the transparency of our model inference process. Since the TCF models incorporated sub-models without feature importance, we weighted feature importance only for the incorporated models that supported feature importance, and detailed results are presented in the Supplementary Table 9. In our study, factors such as duration of mechanical ventilation, number of resuscitations, and other factors included in the “International Guidelines for the Management of Sepsis and Septic Shock: 2016” have been identified by the TCF models as important features for predicting the risk of death from septic shock, which is in line with previous clinical and basic research on sepsis^21,22^. Our predictive variables are all routine clinical variables. In the course of clinical use, if the quality of the variables is poor or unavailable, we strongly recommend that clinicians supplement with additional test results.

This study has several limitations. Firstly, although methods such as correlation analysis and external validation have confirmed that the model is minimally affected by covariate shift, a smaller number of predictive variables would be more user-friendly. Secondly, the model validation cohort, while providing initial insights, is limited by its sample size and the imbalance in sample proportions, particularly the scarcity of positive events, which may affect the robustness of the model’s performance. To address this, we integrated four validation datasets, resulting in a combined sample size of 1421, including 287 positive cases. The analysis yielded an AUC of 0.7705 (95% CI 0.7417-0.7983), suggesting promising discriminatory ability. We also augmented cohorts with insufficient positive samples through oversampling, further demonstrating the reliability of the validation results (Supplementary Table 12). Nevertheless, future could collect enough data for cross-specialty and multi-center validation, ensuring its generalizability and reliability across diverse clinical settings. Thirdly, this study validated the model across three centers, providing preliminary evidence of its performance, further research is needed to explore its generalizability across broader and more diverse regions. Fourthly, this study focused on leveraging structured clinical feature data derived from electronic medical records to develop the predictive model. While this approach provides valuable insights, future research could explore the integration of additional data modalities. Fifthly, this study, designed as a retrospective analysis, provides a foundational understanding of the model’s potential based on historical data. Future research can conduct prospective studies under ethical approval to evaluate the clinical efficacy of the model^23^.

In conclusion, the TCF model demonstrated stable and robust generalization in predicting the risk of death within 28 days in patients with septic shock based on 34 patient characteristics from the electronic medical record. The predictive performance of the model was significantly better than the seven base models, providing a reliable tool for early intervention in septic shock patients. Our TCF model provides a low-cost, easy-to-use, and accurate tool for predicting the risk of death in septic shock patients. Future studies could collect data from more regions for validation and conduct prospective studies to further confirm the feasibility of the TCF model we developed.

## Methods

### Study design and participants

This study was approved by the Institutional Review Board of Tongji Hospital, Tongji Medical College, Huazhong University of Science and Technology (Approval No. TJ-IRB202412029, the three centers were governed by the same ethics review committee), and complied with the Declaration of Helsinki. The ethics committee waived the requirement for informed consent as this retrospective study analyzed only existing, fully anonymized clinical data with no additional patient interventions. All data were de-identified prior to analysis to protect participant privacy. In this multicenter retrospective study, we collected clinical information on 4872 ICU patients from February 2003 to November 2023 from the electronic medical records of three hospitals in China. This retrospective study included patients with septic shock from the intensive care units (ICUs) (Cohort 1, Cohort 2, and Cohort 3). Cohort 1 contained patients from three clinical specialties: general ICU, pediatric ICU, and respiratory ICU. We used 3451 ICU patients from the general ICU (ICU cohort) as the primary study population for model training and internal validation. A total of 357 patients from pediatric ICU (Cohort 1-1) and 381 patients from respiratory ICU (Cohort 1-2) were used as cross-specialty validation data to assess the stability of the model across specialties. In addition, 422 participants from Cohort 2 and 261 participants from Cohort 3 provided multicenter data for this study, representing patients with septic shock from different ICUs, to assess the external generalization performance of the model. Septic shock diagnoses and outcomes (discharge or death) for all participants were confirmed by the attending physician and recorded in the electronic medical record.

**Fig. 7:**
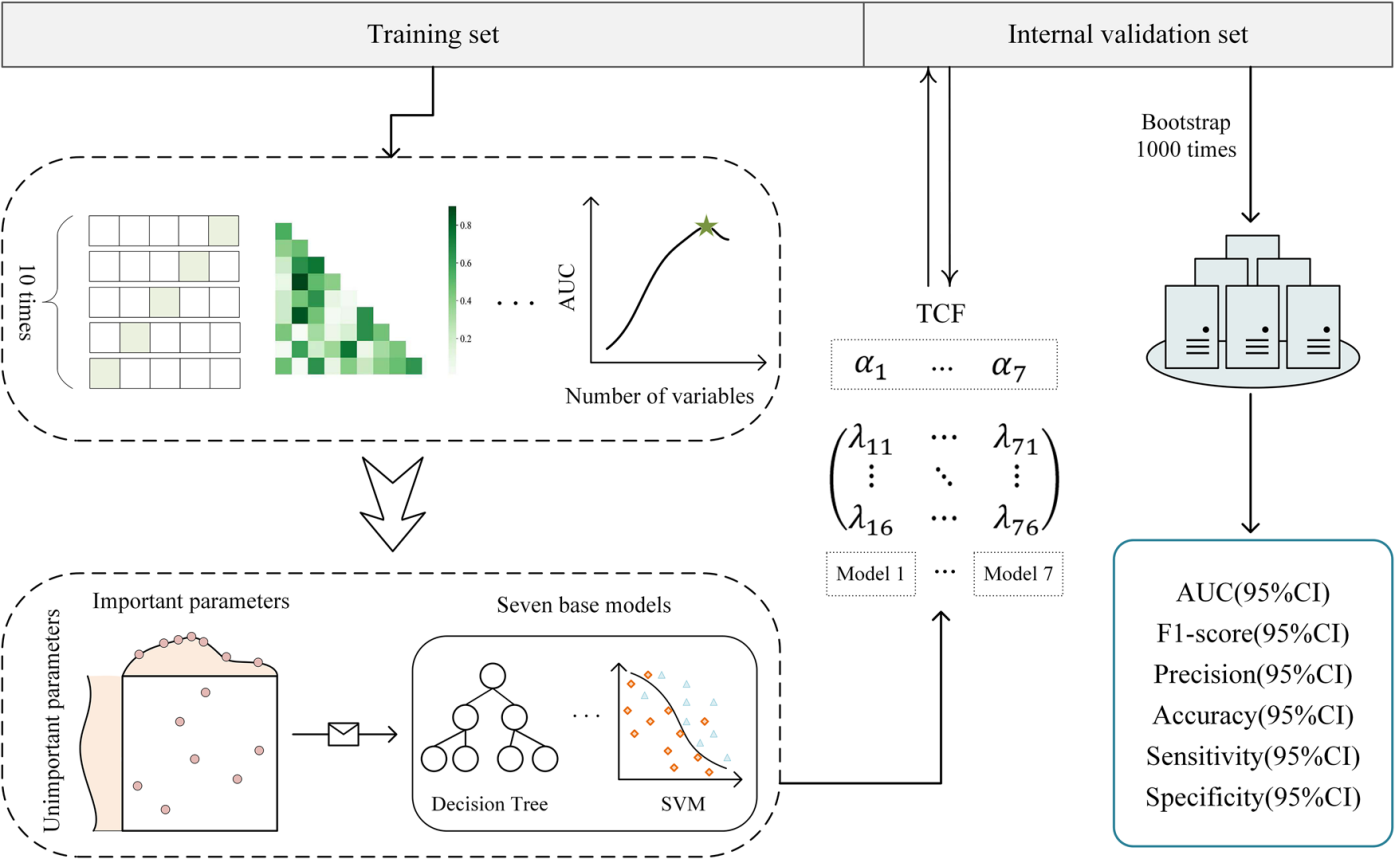
Study design. An illustration of how the predictive model is established based on the electronic health record system.

Our clinically intelligent prediction research is primarily divided into three steps. The first step involved using in-hospital data of patients with infectious shock to establish seven sub-models, each yielding results for six evaluation metrics. The second step was based on a fusion strategy, integrating the sub-models into a comprehensive model (TCF) and demonstrating that the predictive performance of TCF was superior to other models. The third step involved testing the model’s performance across various datasets and conducting an interpretability analysis of the model. The research design adheres to the TRIPOD + AI guidelines^24^ (Supplementary Table 13), as illustrated in Fig. 7. As this was a retrospective study, there was no public involvement, no research protocol was prepared, and the study was not registered.

### Data preprocessing

Based on their universality in routine clinical use by ICU working doctors, 93 intramural indices were screened, including demographics information (e.g., age and gender), disease and treatment history (e.g., chemotherapy history), admission information (e.g., length of stay in the ICU until discharge), laboratory tests (e.g., white blood cell count and lymphocyte count), vital sign information (e.g., height and blood pressure), and medication information (e.g., epinephrine). Participants were measured multiple times for the same item upon admission to the ICU, and laboratory test data from the first measurement were selected for the study. Additionally, records of drug injections or oral intake within 24 h were included in the analysis. Four metrics were obtained through calculation:

2 $${NLR}=\frac{{Neutrophil\; Count}\left({10}^{9}/L\right)}{{Lymphocyte\; count}\left({10}^{9}/L\right)}$$

3 $${PLR}=\frac{{Platelet\; count}\left({10}^{9}/L\right)}{{Lymphocyte\; count}\left({10}^{9}/L\right)}$$

4 $${LMR}=\frac{{Lymphocyte\; count}\left({10}^{9}/L\right)}{{Monocyte\; count}\left({10}^{9}/L\right)}$$

5 $${PAR}=\frac{{Platelet\; count}\left({10}^{9}/L\right)}{{Albumin}\left(g/L\right)}$$

6 $${SI}=\frac{{Heart\; rate}\left({beats\; per\; minute}\right)}{{Systolic\; blood\; pressure}\left({mmHg}\right)}$$

Raw data are difficult to utilize directly; appropriate feature processing can help us better capture participants’ information and build models with better performance. Our feature processing includes five parts: (1) delete features with a missing rate greater than 30%, as features with too high a missing rate lose the value of interpolation; (2) calculate the variance of Boolean features according to Bernoulli’s variance formula and delete features with more than 90% agreement; (3) consider four common missing value interpolation methods (k-Nearest Neighbors Imputer, Multiple Imputation using Logistic Regression, Multiple Imputation using the k-Nearest Neighbors, and Ridge Regression) interpolate the missing values, and then build the same classification model separately to compare the effects and choose the best method as our missing value interpolation strategy; (4) calculate the Pearson correlation coefficients between pairs of features, and for combinations of features with correlations not less than 0.7, retain the features that make the AUC larger; (5) calculate the information entropy of each feature, understanding that the smaller the entropy value, the higher the information content and the higher the value of the feature, and then sort the features according to the information entropy from high to low, gradually increasing the number of features to build the model and selecting the subset of features that maximize AUC. In addition, the data needs to be min-max normalized before modeling or validation.

### Outcomes

A diagnosis of septic shock, either primary or secondary, was determined by the attending physician and recorded on the first page of the case. The length of time between the participants’ admission to the ICU and the occurrence of the outcome (death or survival to discharge) was calculated. The outcome of the study was whether the participant died of all-cause death within 28 days of admission to the ICU. Participants who experienced all-cause death within 28 days from the start of ICU admission were considered to have a positive outcome, while those who did not were considered to have a negative outcome.

### Prediction modeling

It is unrealistic to exhaust all binary classification models; therefore, fusion models that combine results more effectively than a single model represent an efficient and high-quality strategy^25^. The unprocessed ICU cohort was divided into a training set (80%, *n* = 2760) and an internal validation set (20%, *n* = 691). Seven sub-models were trained on the training set after feature processing, the actual effects of the models were tested on the internal validation set, and the parameters of the fusion model were obtained based on these results. Synthetic Minority Over-sampling Technique (SMOTE) was applied to the training set according to the 1:1 rule to mitigate the negative effects of class imbalance^26^. After min-max normalization, the optimal parameter combination was determined by five-fold cross-validation and random search, and seven models (Decision Tree (DT), Random Forest (RF), XGBoost (XGB), LightGBM (LGBM), Naive Bayes (NB), Support Vector Machine (SVM), Gradient Boosted Decision Tree (GBDT)) were trained on the training set. Finally, the predictions were validated using the internal validation data, and the performance of the models was evaluated by area under ROC curve (AUC), F1-score, precision (PRE), accuracy (ACC), sensitivity (SEN), and specificity (SPE).

Each of the seven models has its own strengths and weaknesses across six indices, and we designed a TOPSIS-based Classification Fusion (TCF) model that combines the estimation results from the seven models in order to develop a comprehensive predictive tool for the diagnosis of septic shock. The model was fused using TOPSIS, where each evaluation index was given equal importance^27^. The sub-model weights are calculated by TOPSIS-score, and the weighted predictive probability is the predictive probability of TCF, with 0.5 as the critical value to derive the classification result of TCF.

Use ${{AUC}}_{i}$, ${F1-{socre}}_{i}$, ${{ACC}}_{i}$, ${{PRE}}_{i}$, ${{SEN}}_{i}$, ${{SPE}}_{i}$ $(i=\mathrm{1,2},\ldots ,7)$ to represent the effects of seven models. The larger the evaluation index, the better the model performance, so all indices are uniformly transformed into extreme-type indices, for example, ${{AUC}}_{i}^{^{\prime} }=\max \left({{AUC}}_{i}\right)-{{AUC}}_{i}$. Similarly, get all ${F1-{score}}_{i}^{^{\prime} }$, ${{PRE}}_{i}^{^{\prime} }$, ${{ACC}}_{i}^{^{\prime} }$, ${{SEN}}_{i}^{^{\prime} }$, ${{SPE}}_{i}^{^{\prime} }$ $({\rm{i}}=1,2,\ldots ,7)$. After the matrix is positively transformed, it undergoes a standardization process to eliminate the impact of varying attribute dimensions, thereby ensuring that the values of each index are comparable on the same scale. Take AUC as an example,

7 $${{AUC}}_{i}{\text{'}\text{'}}=\frac{{{AUC}}_{i}^{^{\prime} }}{\sqrt{\sum _{i=1}^{7}{{{AUC}}_{i}^{^{\prime} }}^{2}}},i=1,2,\ldots ,7$$

similarly, get all $F1-{\mathrm{score}}_{i}\text{'}\text{'}$, ${{P}{R}{E}}_{{i}}{\text{'}\text{'}}$, $A{{CC}}_{{i}}{\text{'}\text{'}}$, ${{S}{E}{N}}_{{i}}{\text{'}\text{'}}$, $S{{PE}}_{{i}}{\text{'}\text{'}}$.

Assign the same weights to the indices, the weight vector is$\,\left(1/6,1/6,1/6,1/6,1/6,1/6\right)$. Multiply each value of the normalized decision matrix by its corresponding weight to obtain a weighted normalized matrix, ensuring that the contribution of each scoring index is proportional to its weight. The maximum and minimum values of each index across the seven models are identified to form the sets of positive ideal solution (PIS) and negative ideal solution (NIS), respectively ^28^.

8 $${PIS}=\left({{AUC}}_{\max },{F1}_{\max },{{PRE}}_{\max },{{ACC}}_{\max },{{SEN}}_{\max },{{SPE}}_{\max }\right)$$

9 $${NIS}=\left({{AUC}}_{\min },{F1}_{\min },{{PRE}}_{\min },{{ACC}}_{\min },{{SEN}}_{\min },{{SPE}}_{\min }\right)$$

Calculate the comprehensive evaluation index of each model, the comprehensive evaluation index of the *i-th* model is ${{\rm{score}}}_{i}$,

10 $${{{topsis}}_{{score}}}_{i}=\frac{{scor}{e}_{i,\min }}{{scor}{e}_{i,\min }+{scor}{e}_{i,\max }},i=1,2,\ldots ,7$$

where ${{score}}_{i,\max }$ and ${{score}}_{i,\min }$ are defined as

11 $${{score}}_{i,\max }=\sqrt{{\left({{AUC}}_{\max }-{{A}{U}{C}}_{{i}}{\text{'}\text{'}}\right)}^{2}+{\left({F1}_{\max }-{F1}_{i}\text{'}\text{'}\right)}^{2}+\cdots +{\left({{SPE}}_{\max }-S{{PE}}_{{i}}{\text{'}\text{'}}\right)}^{2}}$$

12 $${{score}}_{i,\min }=\sqrt{{\left({{AUC}}_{\min }-A{{UC}}_{{i}}{\text{'}\text{'}}\right)}^{2}+{\left({F1}_{\min }-{F1}_{i}\text{'}\text{'}\right)}^{2}+\cdots +{\left({{SPE}}_{\min }-S{{PE}}_{{i}}{\text{'}\text{'}}\right)}^{2}}$$

Use ${{{topsis}}_{{score}}}_{i}(i=\mathrm{1,2},\ldots ,7)$ to represent the comprehensive scoring index of DT, RF, XGB, LGBM, SVM, NB, GBDT, define ${\alpha }_{i}\left(i=\mathrm{1,2},\ldots ,7\right)$ as

13 $${\alpha }_{i}=\frac{{{topsi}{s}_{{score}}}_{i}}{\sum {{topsi}{s}_{{score}}}_{i}},i=1,2,\ldots ,7$$

Assuming that we obtain the negative predictive results for $N$ participants through seven models as matrix $M3$,

14 $$M3=\left({p}_{{ij}}\right),i=1,2,...,7;j=1,2,\ldots ,N$$

the positive probability of the *j-th* participant after model fusion is

15 $${p}_{j}=\sum _{i=1}^{7}{\alpha }_{i}{p}_{{ij}},\left(j=1,2,\ldots ,N\right)$$

### Statistical analysis

Statistics on certain key features of the participants are provided to facilitate a quicker understanding of the background of the research subjects. For continuous features, statistics on the median, upper quartile, and lower quartile are presented, and for discrete features, the proportion of each category is reported. In our study, the smallest dataset is cohort3 (*n* = 261). According to the central limit theorem, the distribution of the mean of continuous features can be considered normal. Levene’s test was applied to determine the homogeneity of features between the two sets of data. Chi-square test was employed to compare the differences in discrete features between other data and the internal validation set, while Independent-Sample *t*-test or Welch’s t-test was utilized to assess the differences in continuous features. The 95% confidence intervals for the assessment indices were calculated using 1000 bootstrap samples.

### Feature assessment

TCF obtains a recognized feature priority ranking by calculating the feature importance weights built into the sub-models. SVM focuses on the criticality of decisions, seeking the optimal hyperplane rather than the contribution of all features, and NB assumes that features are independent of each other, considering each feature equally important^29,30^. Therefore, neither of these two types of models can provide feature importance weights. DT, RF, XGB, LGBM, and GBDT output weights for 34 features, respectively, and the TCF feature ranking is obtained by sorting the sum of these weights. Meanwhile, SHapley Additive exPlanation (SHAP) also helps to interpret the sub-models of TCF^31^.

## Supplementary information

Supplementary information

## Data Availability

The data that support the findings of this study are available on request from the corresponding author Q.Y. The data are not publicly available due to them containing information that could compromise research participant privacy.

## Code availability

Data analysis was conducted using SPSS Statistics (version 26) and Python (version 3.10.9). The core code for the research can be found on the corresponding author’s GitHub page (https://github.com/472404420/NPJ-codes).
