# Supplementary material for: Artificial intelligence based multispecialty mortality prediction models for septic shock in a multicenter retrospective study
Source: NPJ Digit Med. 2025 Apr 28;8:228. doi: 10.1038/s41746-025-01643-w (PMC12037723; doi:10.1038/s41746-025-01643-w)
Supplement: Supplementary file 1 — Supplementary information [file 41746_2025_1643_MOESM1_ESM.pdf]

Supplementary Information

Artificial Intelligence Based Multispecialty Mortality Prediction Models for Septic Shock in a Multicenter Retrospective Study

Contents:

**Supplementary item**	**Page**
Supplementary figures	2
Supplementary Fig. 1 | Feature processing flow	2
Supplementary Fig. 2 | Correlation coefficient heatmap	3
Supplementary Fig. 3 | The calibration curves of all models on the internal validation set	4
Supplementary Fig. 4 | The DCA result of TCF model on the internal validation set	5
Supplementary Fig. 5 | SHAP feature summary plot of the optimal sub-model	6
Supplementary Fig. 6 | The calibration curves on the validation set	7
Supplementary Fig. 7 | The DCA result of TCF model on the validation set	8
Supplementary tables	9
Supplementary Table 1 | The hypothesis testing results for cohort 1-1, cohort 1-2, cohort 2, and cohort 3	9
Supplementary Table 2 | Comparison of three highly correlated variable combinations	11
Supplementary Table 3 | Results of pairwise comparisons of highly correlated variables	12
Supplementary Table 4 | Comparison of results from different methods of imputing missing values	13
Supplementary Table 5 | Stepwise inclusion of variables based on entropy ranking for modeling	14
Supplementary Table 6 | Hyperparameter tuning information for various models on the training set	15
Supplementary Table 7 | Evaluation of classification performance for seven sub-models	16
Supplementary Table 8 | Evaluation of the classification performance of the TCF model	17
Supplementary Table 9 | TCF feature importance ranking	18
Supplementary Table 10 | Performance of the TCF across different datasets	19
Supplementary Table 11 | The missing rate of the training dataset	20
Supplementary Table 12 | Validation results after oversampling the validation data	21
Supplementary Table 13 | TRIPOD+AI guidelines	22

Supplementary Fig. 1 | Feature processing flowchart. The detailed process of feature handling in this study can be represented by a flowchart, which includes a series of feature engineering steps such as missing rate filtering, variance filtering, missing value imputation, correlation filtering, and stepwise feature selection.

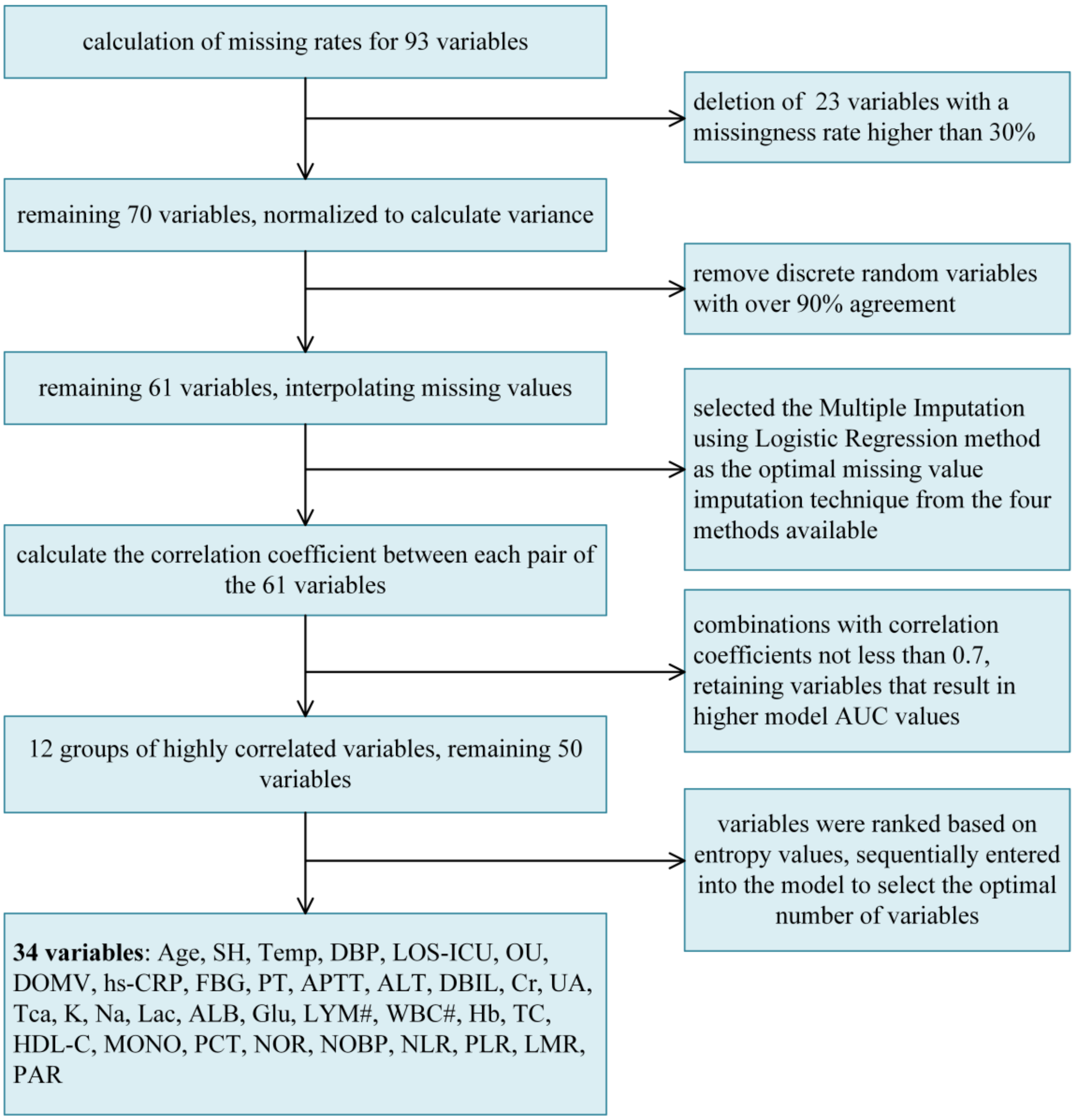

Supplementary Fig. 2 | Correlation coefficient heatmap. A heatmap was generated based on the Pearson correlation coefficients of the 34 variables included in the model, with darker colors indicating higher correlations. The heatmap revealed that all correlations among these variables were below 0.7, indicating no strong correlations.

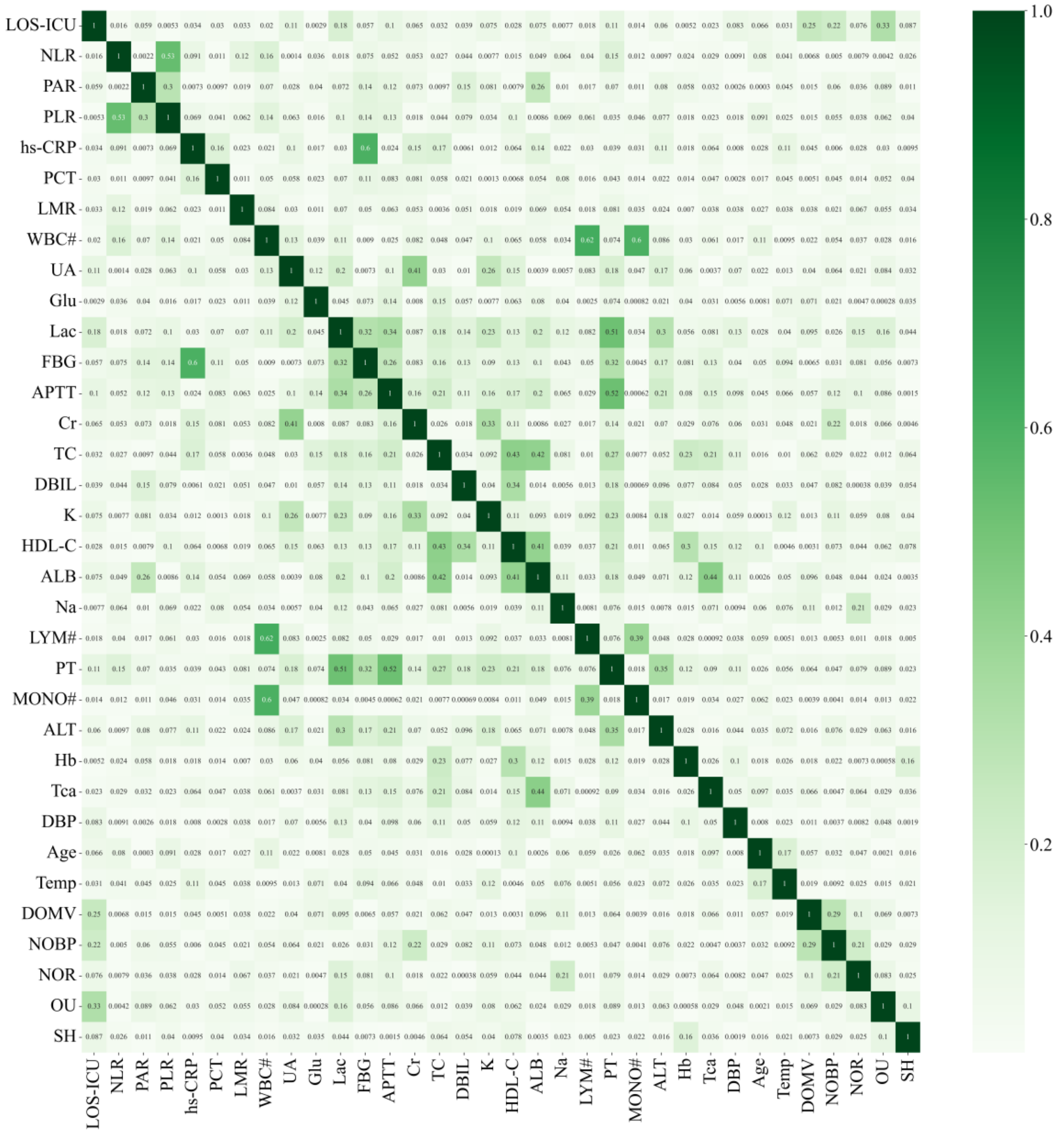

Supplementary Fig. 3 | The calibration curves of all models on the internal validation set. The calibration curve on the internal validation set showed that the TCF model was closest to the diagonal line, indicating it had the best calibration performance among all models.

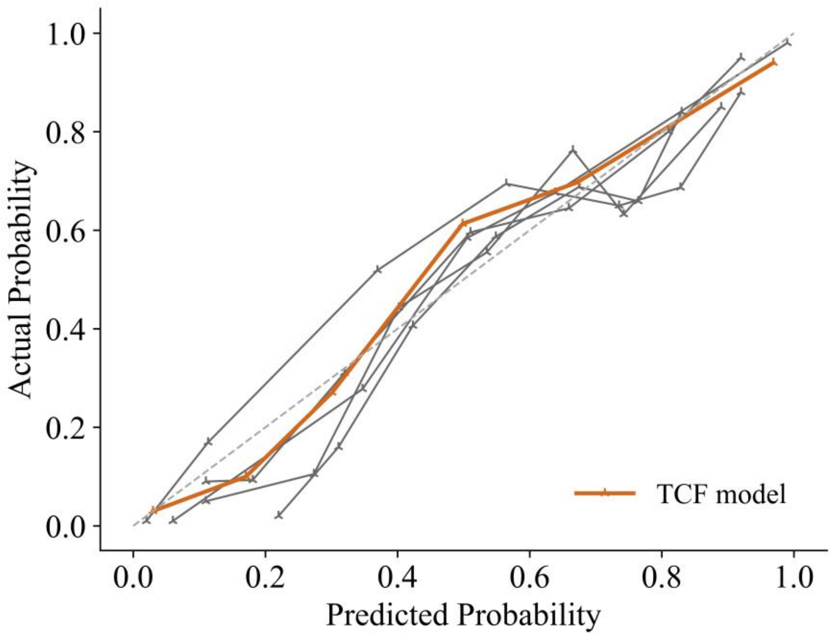

Supplementary Fig. 4 | The DCA result of TCF model on the internal validation set. The TCF model's curve consistently outperforms both the "All" and "None" strategies across most threshold probabilities, demonstrating superior net benefit particularly within the 0.1 to 0.5 probability range.

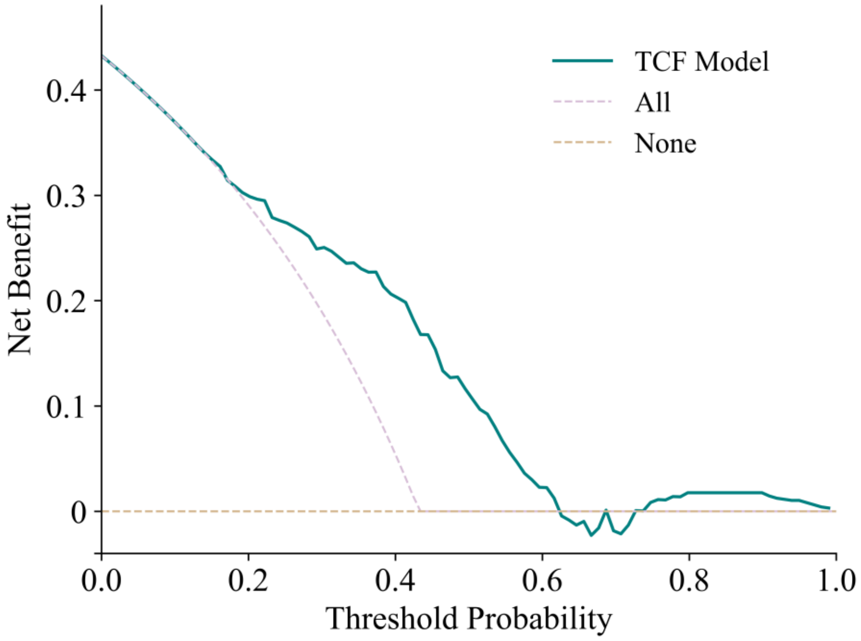

Supplementary Fig. 5 | SHAP feature summary plot of the optimal sub-model. The SHAP feature importance analysis of the optimal sub-model (GBDT model) visualized the impact of different features on model outputs. Features were sorted by importance, with the most influential ones positioned at the top. Key predictive features included the number of resuscitations, duration of mechanical ventilation, ICU length of stay, among others, all demonstrating significant contributions to the model's performance.

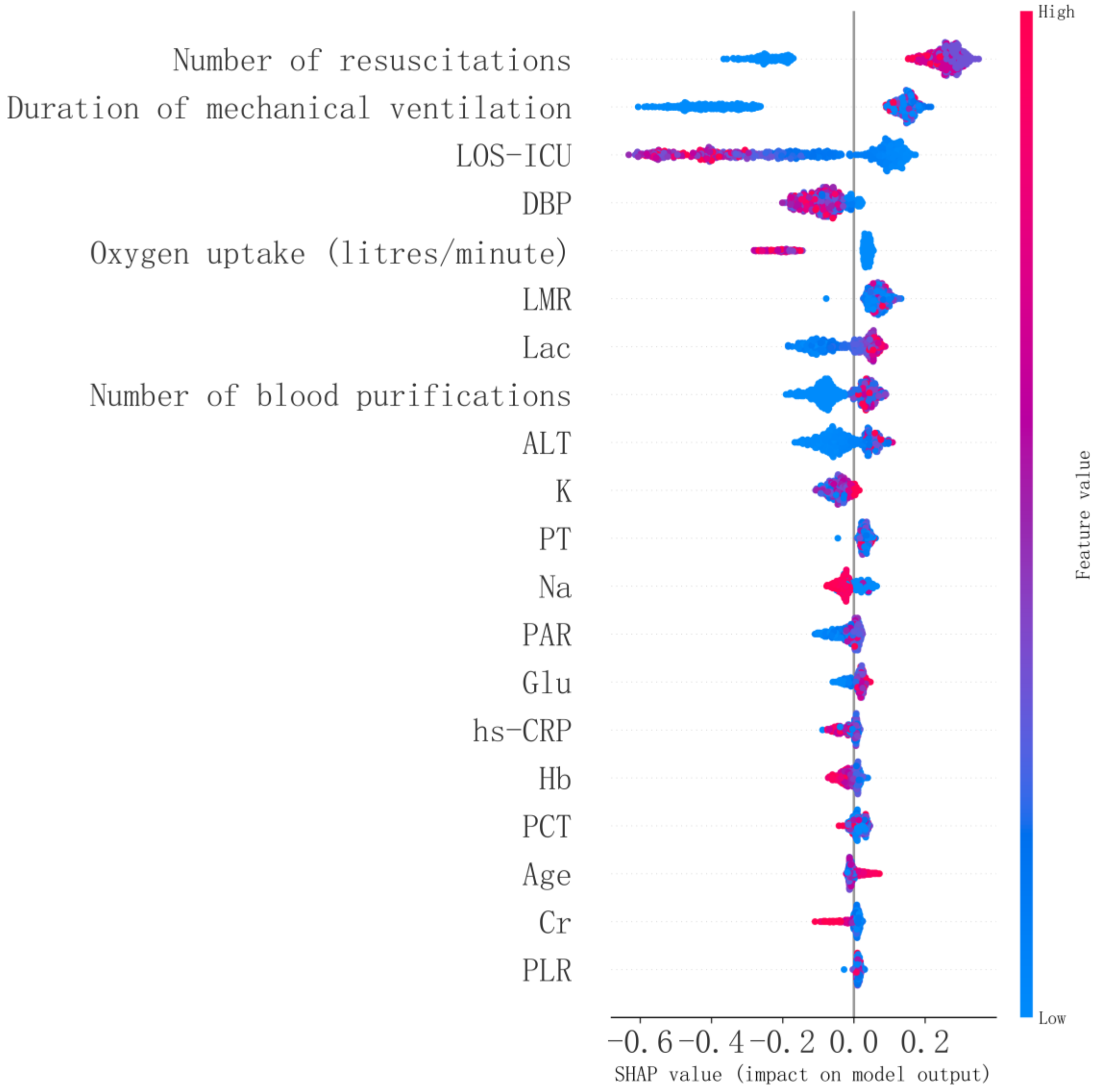

Supplementary Fig. 6 | The calibration curves on the validation set. The curves plotted after merging the prediction results of cohort 1-1, cohort 1-2, cohort 2, and cohort 3 using the TCF model.

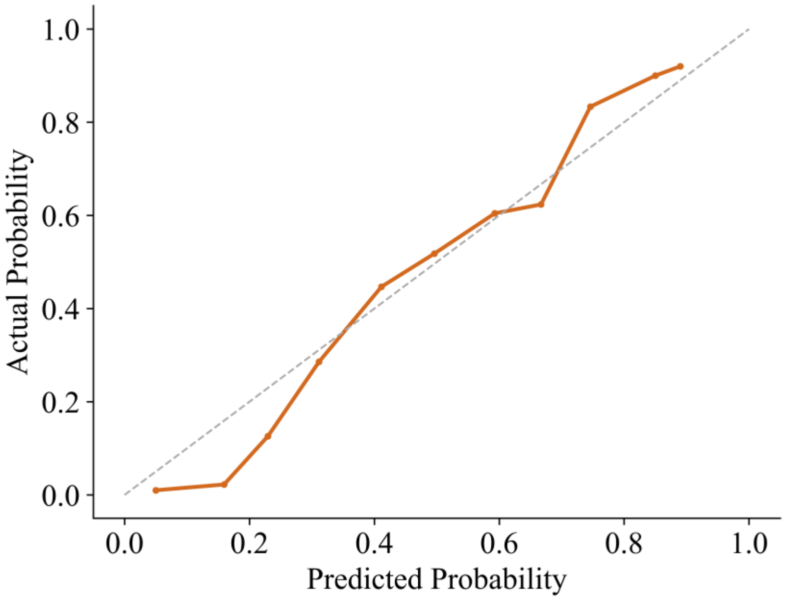

Supplementary Fig. 7 | The DCA result of TCF model on the validation set. The curves plotted after merging the prediction results of cohort 1-1, cohort 1-2, cohort 2, and cohort 3 using the TCF model.

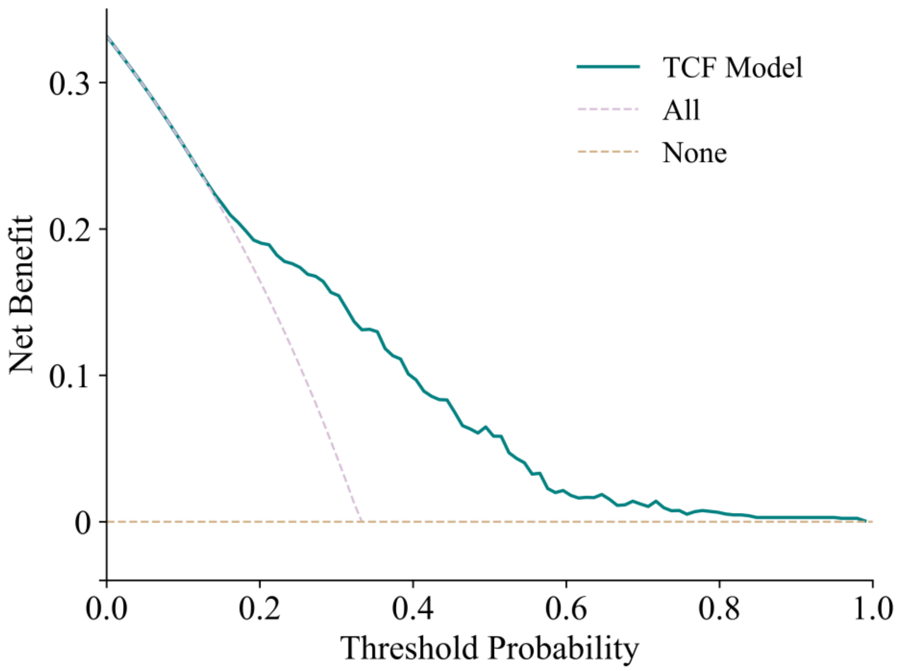

Supplementary Table 1 | The hypothesis testing results for cohort 1-1, cohort 1-2, cohort 2, and cohort 3. In our study, the smallest dataset is cohort3 (n=261), according to the central limit theorem, the distribution of the mean of continuous features can be considered normal. Levene's test was applied to determine the homogeneity of features between the two sets of data. Independent-Sample t-test or Welch's t-test was utilized to assess the differences in continuous features.

	**Cohort 1-1**				**Cohort 1-2**				**Cohort 2**				**Cohort 3**			
	Levene's Test		t-test chi-square test		Levene's Test		t-test chi-square test		Levene's Test		t-test chi-square test		Levene's Test		t-test chi-square test	
	F-value	p-value	t-value	p-value	F-value	p-value	t-value	p-value	F-value	p-value	t-value	p-value	F-value	p-value	t-value	p-value
SH	/	/	48.420	<0.05	/	/	3.342	0.068	/	/	0.479	0.489	/	/	2.566	0.109
LOS-ICU	1.319	0.251	-5.834	0.000	12.207	0.000	-5.012	0.000	0.475	0.491	-2.340	0.019	12.089	0.001	2.195	0.029
NLR	124.576	0.000	16.266	0.000	0.130	0.718	0.959	0.338	2.425	0.120	-1.112	0.266	4.371	0.037	-0.918	0.359
PAR	4.106	0.043	-4.557	0.000	0.817	0.366	-3.351	0.001	0.931	0.335	-1.025	0.305	1.614	0.204	0.053	0.957
PLR	4.363	0.037	2.685	0.007	0.712	0.399	-2.150	0.032	11.507	0.001	-2.782	0.006	0.405	0.524	-1.313	0.189
hs-CRP	12.712	0.000	8.380	0.000	15.740	0.000	3.171	0.002	0.203	0.653	1.655	0.098	0.509	0.476	0.321	0.748
PCT	81.154	0.000	5.879	0.000	175.742	0.000	10.053	0.000	1.040	0.308	0.739	0.460	0.312	0.577	-0.478	0.633
LMR	54.167	0.000	-6.444	0.000	27.439	0.000	3.402	0.001	7.538	0.006	2.303	0.021	11.225	0.001	2.977	0.003
WBC#	0.977	0.323	0.596	0.552	26.271	0.000	2.252	0.025	0.018	0.892	-0.797	0.426	1.342	0.247	0.328	0.743
UA	1.218	0.270	4.034	0.000	2.555	0.110	3.856	0.000	0.083	0.773	-1.208	0.227	1.862	0.173	-3.517	0.000
Glu	34.675	0.000	-0.166	0.868	0.770	0.380	-0.589	0.556	6.862	0.009	-2.119	0.034	1.240	0.266	-0.484	0.629
Lac	19.152	0.000	0.713	0.476	2.772	0.096	1.738	0.083	9.818	0.002	3.124	0.002	3.754	0.053	1.415	0.158
FBG	1.740	0.187	1.882	0.060	11.922	0.001	-5.909	0.000	8.240	0.004	-1.970	0.049	2.324	0.128	-2.866	0.004
APTT	14.925	0.000	2.972	0.003	45.869	0.000	8.696	0.000	2.064	0.151	4.901	0.000	7.185	0.007	3.391	0.001

Cr	175.191	0.000	18.193	0.000	40.431	0.000	6.587	0.000	1.617	0.204	0.607	0.544	0.649	0.421	-1.986	0.047
TC	2.593	0.108	-4.592	0.000	0.043	0.836	-8.869	0.000	0.383	0.536	-6.409	0.000	0.515	0.473	-4.738	0.000
DBIL	38.453	0.000	5.970	0.000	77.905	0.000	7.498	0.000	8.654	0.003	2.647	0.008	14.306	0.000	3.091	0.002
K	4.722	0.030	2.324	0.020	11.385	0.001	3.729	0.000	1.512	0.219	2.909	0.004	0.274	0.601	1.661	0.097
HDL-C	3.935	0.048	-7.887	0.000	1.808	0.179	-4.446	0.000	1.016	0.314	-7.365	0.000	2.210	0.138	-4.963	0.000
ALB	2.316	0.128	-16.084	0.000	2.919	0.088	-10.700	0.000	1.677	0.196	-8.092	0.000	2.275	0.132	-6.257	0.000
Na	189.551	0.000	-4.178	0.000	14.634	0.000	-1.049	0.294	5.660	0.018	-0.293	0.769	852.278	0.000	-12.550	0.000
LYM#	12.340	0.000	-2.636	0.009	0.737	0.391	-0.727	0.468	6.727	0.010	-1.234	0.218	1.974	0.160	0.877	0.381
PT	11.542	0.001	4.582	0.000	36.142	0.000	8.012	0.000	5.648	0.018	4.315	0.000	10.509	0.001	4.205	0.000
MONO	4.415	0.036	-1.674	0.095	1.731	0.189	-0.345	0.730	13.459	0.000	-1.452	0.147	0.026	0.871	-0.930	0.353
ALT	4.373	0.037	1.214	0.225	19.876	0.000	3.181	0.002	2.145	0.143	1.019	0.308	0.159	0.690	-0.205	0.837
Hb	29.602	0.000	1.777	0.076	19.079	0.000	0.019	0.985	2.417	0.120	-2.702	0.007	0.081	0.776	-0.541	0.589
Tca	0.967	0.326	-13.042	0.000	3.805	0.051	-9.379	0.000	0.463	0.496	-4.757	0.000	0.193	0.661	-4.880	0.000
DBP	2.083	0.149	7.581	0.000	14.612	0.000	-5.357	0.000	0.487	0.486	-1.678	0.094	1.795	0.181	-0.761	0.447
Age	317.677	0.000	78.159	0.000	0.188	0.664	-3.863	0.000	3.171	0.075	-4.355	0.000	5.445	0.020	-5.383	0.000
Temp	7.181	0.007	-7.016	0.000	9.430	0.002	-1.877	0.061	3.960	0.047	-0.324	0.746	2.065	0.151	1.153	0.249
DOMV	9.791	0.002	3.294	0.001	38.964	0.000	-2.889	0.004	4.767	0.029	2.265	0.024	10.880	0.001	2.758	0.006
NOBP	45.263	0.000	6.587	0.000	1.035	0.309	1.920	0.055	1.375	0.241	0.146	0.884	7.132	0.008	-1.955	0.051
NOR	340.161	0.000	-14.943	0.000	1.979	0.160	10.855	0.000	0.193	0.660	1.263	0.207	0.899	0.343	1.033	0.302
OU	128.776	0.000	6.374	0.000	37.357	0.000	3.515	0.000	6.506	0.011	0.939	0.348	3.492	0.062	-1.191	0.234

Supplementary Table 2 | Comparison of three highly correlated variable combinations. The pairwise correlation coefficients among SBP, DBP, and MAP exceeded 0.7, forming a strongly correlated variable group. Consequently, only one variable from this group could be retained in the final model. We systematically evaluated all three possible retention scenarios (keeping either SBP, DBP, or MAP) and selected the option that maximized the AUC.

**Delete variables**		**AUC**	**Final scheme**
SBP	DBP	0.845	
SBP	MAP	0.877	√
DBP	MAP	0.844	

Supplementary Table 3 | Results of pairwise comparisons of highly correlated variables. For variable groups with correlation coefficients above 0.7, two potential elimination approaches were tested, retaining the variable that yielded a higher AUC.

**Variable_1**	**Variable_2**	**AUC**		**Final retained variables**
		**Retention of variable _1**	**Retention of variable _2**	
PT-INR	PT	0.823	0.845	PT
ALT	AST	0.847	0.843	ALT
TBIL	DBIL	0.828	0.850	DBIL
ALB	TP	0.849	0.840	ALB
WBC#	NEU#	0.856	0.835	WBC#
D-Dimer	PCT	0.830	0.833	PCT
PLT#	PAR	0.825	0.852	PAR
TC	LDL-C	0.859	0.834	TC
Gender	Pregnancy history	0.830	0.849	Pregnancy history

Supplementary Table 4 | Comparison of results from different methods of imputing missing values. Combining cross-validation, we evaluated four missing value imputation methods on the training set, with Multiple Imputation using Logistic Regression proving to be the optimal choice.

Method	AUC
k-Nearest Neighbors Imputer	0·829
Multiple Imputation using Logistic Regression	0·844
Multiple Imputation using the k-Nearest Neighbors Algorithm	0·826
Ridge Regression	0·805

Supplementary Table 5 | Stepwise inclusion of variables based on entropy ranking for modeling. The features were prioritized for model inclusion based on their information entropy ranking, with progressively more variables added while tracking the corresponding AUC values.

Top n variables	AUC	Top n variables	AUC
2	0.6398	28	0.7365
4	0.6577	30	0.7718
6	0.6759	32	0.8123
8	0.66996	34	0.8410
10	0.6829	36	0.8310
12	0.6856	38	0.8132
14	0.6864	40	0.8225
16	0.6880	42	0.8271
18	0.7012	44	0.8211
20	0.7165	46	0.8137
22	0.6762	48	0.8270
24	0.7048	50	0.8254
26	0.6757		

Supplementary Table 6 | Hyperparameter tuning information for various models on the training set. All model parameters in the study were obtained through random search. Below are the specified parameter ranges and the optimal parameter results.

Model	Parameters	Range	Optimum value
DT	criterion	['entropy', 'gini']	gini
	max_depth	[10, 20, 30, …, 300]	200
	max_features	['sqrt', 'log2']	sqrt
	max_leaf_nodes	[10, 20, 30, …, 100]	40
	min_samples_split	[10, 20, 30, …, 100]	10
	splitter	['best', 'random']	best
	min_samples_leaf	[10, 20,30, …, 100]	70
RF	n_estimators	[20, 25, 30, …, 100]	75
	criterion	['gini', 'entropy']	gini
	max_depth	[10, 20, 30, …, 100]	20
	min_samples_leaf	[10, 20, 30, …, 100]	10
	min_samples_split	[10, 20, 30, …, 100]	10
	max_features	['sqrt', 'log2']	sqrt
XGB	max_depth	[20, 25, 30, …, 50]	20
	min_child_weight	[5, 6, 7,…, 20]	16
	lambda	[10, 15, 20, …,50]	20
	subsample	[0.5, 0.6, 0.7, 0.8, 0.9, 1]	0.9
	colsample_bytree	[0.5, 0.6, 0.7, 0.8, 0.9, 1]	0.6
	eta	[0.01, 0.02, 0.03, …, 0.1]	0.06
	gamma	[0.001, 0.005, 0.01, 0.05, 0.1]	0.01
	n_estimators	[20, 25, 30, …, 50]	30
LGBM	boosting_type	['gbdt', 'goss', 'dart', 'rf']	gbdt
	num_leaves	[5, 10, 15, …, 50]	45
	max_depth	[10, 15, 20, …, 50]	25
	learning_rate	[0.001, 0.002, 0.003, …, 0.050]	0.031
	n_estimators	[10, 15, 20, …, 50]	35
	subsample	[0.1, 0.2, 0.3, …, 1]	0.2
	colsample_bytree	[0.1, 0.2, 0.3, …, 1]	0.4
	reg_alpha	[0.01, 0.05, 0.1, 0.5 ,1]	0.01
SVM	C	[0.01, 0.02, 0.03, …, 0.5]	0.25
	gamma	[0.01, 0.02, 0.03, …, 0.5]	0.4
	kernel	['linear', 'rbf', 'poly']	rbf
	degree	[1, 2, 3, …,10]	5
NB	alpha	[0.1, 0.2, 0.3, …, 0.9]	0.3
	binarize	[0.0, 0.1, 0.2, …, 1.0]	0
GBDT	n_estimators	[10, 20, 30, …, 100]	90
	learning_rate	[0.01, 0.02, 0.03, …, 0.1]	0.01
	max_depth	[2, 3, 4, …, 20]	8
	subsample	[0.4,0.5,0.6,0.7,0.8]	0.6
	min_samples_split	[2, 4, 6, …, 20]	4
	min_samples_leaf	[20, 25, 30, …, 50]	20
	max_features	['sqrt', 'log2']	sqrt

Supplementary Table 7 | Evaluation of classification performance for seven sub-models. The performance of seven submodels on the training set was evaluated using six metrics, with 95% confidence intervals derived from 1000 bootstrap samples.

	**AUC**	**F1-score**	**ACC**	**PRE**	**SEN**	**SPE**
DT	0.869 (0.859-0.880)	0.796 (0.783-0.810)	0.790 (0.778-0.802)	0.771 (0·754-0·789)	0.823 (0.807-0.838)	0.756 (0.739-0.775)
RF	0.985 (0.982-0.987)	0.928 (0.920-0.935)	0.928 (0.920-0.935)	0.923 (0.912-0.934)	0.933 (0.922-0.944)	0.922 (0.911-0.933)
XGB	0.948 (0.942-0.954)	0.877 (0.866-0.887)	0.877 (0.866-0.887)	0.874 (0.861-0.888)	0.880 (0.866-0.894)	0.874 (0.859-0.887)
LGB	0.982 (0.979-0.985)	0.933 (0.924-0.940)	0.934 (0.926-0.941)	0.951 (0.942-0.961)	0.915 (0.903-0.927)	0.953 (0.944-0.962)
SVM	0·845 (0·833-0·856)	0.778 (0.765-0.790)	0.762 (0.750-0.773)	0.728 (0.711-0.745)	0.835 (0.821-0.849)	0·688 (0·670-0·707)
NB	0.825 (0.813-0.837)	0.786 (0.774-0.798)	0.766 (0.754-0.778)	0.723 (0.707-0.740)	0·861 (0·846-0·875)	0.671 (0.652-0.689)
GBDT	0.964 (0.959-0.968)	0.892 (0.883-0.901)	0.890 (0.882-0.899)	0.877 (0.863-0.889)	0.909 (0.897-0.921)	0.872 (0.858-0.885)

Supplementary Table 8 | Evaluation of the classification performance of the TCF model. The TOPSIS method was employed to integrate the computational results from seven sub-models, incorporating the following key components: Positive Ideal Solution (PIS), Negative Ideal Solution (NIS), Comprehensive scoring index (α_i), Final ranking, and model coefficients.

	PIS	NIS	α_i	Ranking	Ranking_min_max
DT	0.070	0.100	0.588	6	0.141
RF	0.066	0.116	0.638	4	0.153
XGB	0.070	0.129	0.647	2	0.155
LGBM	0.080	0.136	0.629	5	0.151
SVM	0.136	0.080	0.370	7	0.089
NB	0.074	0.131	0.640	3	0.153
GBDT	0.060	0.114	0.658	1	0.158

Supplementary Table 9 | TCF feature importance ranking. The built-in feature importance scores from tree-based models were ranked and aggregated to derive composite feature importance metrics.

Variable	DT	RF	XGB	LGBM	GBDT	SUM	Rank
DOMV	0.627	0.210	0.302	0.049	0.210	1.398	1
NOR	0.048	0.152	0.119	0.082	0.194	0.595	2
LOS-ICU	0.044	0.083	0.055	0.051	0.099	0.332	3
OU	0.045	0.039	0.160	0.014	0.052	0.310	4
NOBP	0.000	0.065	0.059	0.106	0.076	0.307	5
Lac	0.100	0.040	0.029	0.032	0.034	0.235	6
ALT	0.017	0.040	0.023	0.023	0.032	0.136	7
Na	0.026	0.021	0.020	0.035	0.022	0.124	8
LMR	0.021	0.021	0.010	0.028	0.023	0.102	9
PLR	0.012	0.013	0.010	0.031	0.011	0.078	10
PT	0.004	0.018	0.010	0.027	0.014	0.073	11
Age	0.002	0.017	0.009	0.027	0.014	0.069	12
hs-CRP	0.005	0.014	0.011	0.027	0.011	0.067	13
PCT	0.000	0.015	0.008	0.033	0.010	0.067	14
TC	0.002	0.012	0.008	0.035	0.009	0.066	15
Cr	0.006	0.016	0.010	0.021	0.013	0.065	16
DBP	0.000	0.015	0.017	0.018	0.014	0.064	17
Hb	0.003	0.012	0.010	0.030	0.010	0.064	18
Temp	0.011	0.011	0.007	0.028	0.007	0.064	19
Glu	0.000	0.017	0.007	0.026	0.012	0.062	20
PAR	0.001	0.013	0.008	0.026	0.013	0.061	21
UA	0.006	0.012	0.011	0.019	0.011	0.060	22
DBIL	0.004	0.018	0.008	0.012	0.017	0.060	23
K	0.001	0.015	0.008	0.023	0.011	0.059	24
HDL-C	0.004	0.013	0.014	0.016	0.011	0.058	25
NLR	0.001	0.011	0.006	0.027	0.009	0.055	26
ALB	0.001	0.012	0.008	0.025	0.008	0.055	27
Tca	0.004	0.012	0.011	0.018	0.010	0.054	28
APTT	0.000	0.014	0.005	0.021	0.008	0.047	29
LYM#	0.000	0.009	0.008	0.022	0.006	0.046	30
MONO	0.003	0.010	0.009	0.016	0.008	0.045	31
WBC#	0.001	0.011	0.005	0.021	0.008	0.045	32
FBG	0.001	0.014	0.004	0.013	0.009	0.041	33
SH	0.000	0.004	0.012	0.018	0.005	0.038	34

Supplementary Table 10 | Performance of the TCF across different datasets. The TCF model's performance was comprehensively compared across six distinct datasets (training set, internal validation set, cohort 1-1, cohort 1-2, cohort 2, cohort 3), with 95% confidence intervals derived from 1000 bootstrap samples.

	**AUC**	**F1-score**	**ACC**	**PRE**	**SEN**	**SPE**
Training set	0.955 (0.949, 0.960)	0.874 (0.865, 0.884)	0.869 (0.860, 0.879)	0.845 (0.831, 0.860)	0.905 (0.893, 0.917)	0.834 (0.820, 0.849)
Internal validation set	0.733 (0.694, 0.774)	0.458 (0.398, 0.520)	0.686 (0.654, 0.721)	0.358 (0.302, 0.419)	0.640 (0.557, 0.712)	0.700 (0.663, 0.736)
Cohort 1-1	0.808 (0.740, 0.864)	0.510 (0.391, 0.620)	0.863 (0.824, 0.896)	0.521 (0.385, 0.653)	0.500 (0.372, 0.638)	0.922 (0.893, 0.950)
Cohort 1-2	0.662 (0.598, 0.730)	0.214 (0.111, 0.317)	0.790 (0.748, 0.835)	0.262 (0.133, 0.400)	0.182 (0.091, 0.286)	0.904 (0.872, 0.937)
Cohort 2	0.784 (0.733, 0.825)	0.482 (0.387, 0.557)	0.758 (0.716, 0.799)	0.490 (0.381, 0.583)	0.471 (0.373, 0.567)	0.847 (0.806, 0.885)
Cohort 3	0.786 (0.725, 0.840)	0.497 (0.388, 0.596)	0.716 (0.659, 0.770)	0.500 (0.386, 0.619)	0.494 (0.375, 0.614)	0.802 (0.747, 0.862)

Supplementary Table 11 | The missing rate of the training dataset. Missing rate of the 34 features included in the model.

Number	Feature	Missing rate (%)	Number	Feature	Missing rate (%)
1	Age	0	18	LMR	4·60
2	SH	0	19	Na	4·64
3	OU	0	20	Tca	4·67
4	DOMV	0	21	NLR	4·67
5	NOR	0	22	PAR	4·67
6	NOBP	0	23	APTT	5·04
7	LOS-ICU	1·20	24	DBIL	5·04
8	Hb	3·55	25	ALT	5·18
9	WBC#	3·59	26	TC	5·29
10	PT	3·99	27	UA	5·80
11	FBG	4·13	28	Temp	8·01
12	MONO	4·42	29	DBP	11·49
13	Cr	4·49	30	PCT	21·59
14	ALB	4·49	31	hs-CRP	21·92
15	LYM#	4·49	32	Lac	22·36
16	PLR	4·53	33	Glu	23·73
17	K	4·60	34	HDL-C	26·34

Supplementary Table 12 | Validation results after oversampling the validation data. For datasets with fewer than 100 positive samples, resampling was performed. The validation results of the TCF model before and after resampling were then systematically compared.

Cohort	Sample Size (No. of Positives)	AUC	
		Before Sampling	After Sampling
Cohort 1-1	357 (52)	0.808 (0.740-0.864)	0.843 (0.799-0.884)
Cohort 1-2	381 (60)	0.662 (0.598-0.730)	0.716 (0.660-0.765)
Cohort 3	261 (75)	0.786 (0.725-0.840)	0.806 (0.758-0.855)

Supplementary Table 13 | TRIPOD+AI guidelines. This study underwent evaluation using the TRIPOD+AI checklist, with unchecked items deemed inapplicable to the current research.

TRIPOD+AI

Version: 11-January-2024

Section/Topic	Item	Development / evaluation[1]	Checklist item	Reported on page
**TITLE**				
Title	1	D;E	Identify the study as developing or evaluating the performance of a multivariable prediction model, the target population, and the outcome to be predicted	✓
**ABSTRACT**				
Abstract	2	D;E	See TRIPOD+AI for Abstracts checklist	✓
**INTRODUCTION**				
Background	3a	D;E	Explain the healthcare context (including whether diagnostic or prognostic) and rationale for developing or evaluating the prediction model, including references to existing models	✓
	3b	D;E	Describe the target population and the intended purpose of the prediction model in the context of the care pathway, including its intended users (e.g., healthcare professionals, patients, public)	✓
	3c	D;E	Describe any known health inequalities between sociodemographic groups	✓
Objectives	4	D;E	Specify the study objectives, including whether the study describes the development or validation of a prediction model (or both)	✓
**METHODS**				
Data	5a	D;E	Describe the sources of data separately for the development and evaluation datasets (e.g., randomised trial, cohort, routine care or registry data), the rationale for using these data, and representativeness of the data	✓
	5b	D;E	Specify the dates of the collected participant data, including start and end of participant accrual; and, if applicable, end of follow-up	✓
Participants	6a	D;E	Specify key elements of the study setting (e.g., primary care, secondary care, general population) including the number and location of centres	✓
	6b	D;E	Describe the eligibility criteria for study participants	✓
	6c	D;E	Give details of any treatments received, and how they were handled during model development or evaluation, if relevant	✓
Data preparation	7	D;E	Describe any data pre-processing and quality checking, including whether this was similar across relevant sociodemographic groups	✓
Outcome	8a	D;E	Clearly define the outcome that is being predicted and the time horizon, including how and when assessed, the rationale for choosing this outcome, and whether the method of outcome assessment is consistent across sociodemographic groups	✓
	8b	D;E	If outcome assessment requires subjective interpretation, describe the qualifications and demographic characteristics of the outcome assessors	✓
	8c	D;E	Report any actions to blind assessment of the outcome to be predicted	✓
Predictors	9a	D	Describe the choice of initial predictors (e.g., literature, previous models, all available predictors) and any pre-selection of predictors before model building	✓
	9b	D;E	Clearly define all predictors, including how and when they were measured (and any actions to blind assessment of predictors for the outcome and other predictors)	✓
	9c	D;E	If predictor measurement requires subjective interpretation, describe the qualifications and demographic characteristics of the predictor assessors	✓
Sample size	10	D;E	Explain how the study size was arrived at (separately for development and evaluation), and justify that the study size was sufficient to answer the research question. Include details of any sample size calculation	✓
Missing data	11	D;E	Describe how missing data were handled. Provide reasons for omitting any data	✓
Analytical methods	12a	D	Describe how the data were used (e.g., for development and evaluation of model performance) in the analysis, including whether the data were partitioned, considering any sample size requirements	✓
	12b	D	Depending on the type of model, describe how predictors were handled in the analyses (functional form, rescaling, transformation, or any standardisation).	✓
	12c	D	Specify the type of model, rationale[2], all model-building steps, including any hyperparameter tuning, and method for internal validation	✓
	12d	D;E	Describe if and how any heterogeneity in estimates of model parameter values and model performance was handled and quantified across clusters (e.g., hospitals, countries). See TRIPOD-Cluster for additional considerations[3]	✓
	12e	D;E	Specify all measures and plots used (and their rationale) to evaluate model performance (e.g., discrimination, calibration, clinical utility) and, if relevant, to compare multiple models	✓
	12f	E	Describe any model updating (e.g., recalibration) arising from the model evaluation, either overall or for particular sociodemographic groups or settings	✓
	12g	E	For model evaluation, describe how the model predictions were calculated (e.g., formula, code, object, application programming interface)	✓
Class imbalance	13	D;E	If class imbalance methods were used, state why and how this was done, and any subsequent methods to recalibrate the model or the model predictions	✓
Fairness	14	D;E	Describe any approaches that were used to address model fairness and their rationale	✓
Model output	15	D	Specify the output of the prediction model (e.g., probabilities, classification). Provide details and rationale for any classification and how the thresholds were identified	✓

[1] D=items relevant only to the development of a prediction model; E=items relating solely to the evaluation of a prediction model; D;E=items applicable to both the development and evaluation of a prediction model
[2] Separately for all model building approaches.
[3] TRIPOD-Cluster is a checklist of reporting recommendations for studies developing or validating models that explicitly account for clustering or explore heterogeneity in model performance (eg, at different hospitals or centres). Debray et al, BMJ 2023; 380: e071018 [DOI: 10.1136/bmj-2022-071018]

Page 1 of 2

Training versus evaluation	16	D;E	Identify any differences between the development and evaluation data in healthcare setting, eligibility criteria, outcome, and predictors	✓
Ethical approval	17	D;E	Name the institutional research board or ethics committee that approved the study and describe the participant-informed consent or the ethics committee waiver of informed consent	✓
**OPEN SCIENCE**				
Funding	18a	D;E	Give the source of funding and the role of the funders for the present study	✓
Conflicts of interest	18b	D;E	Declare any conflicts of interest and financial disclosures for all authors	✓
Protocol	18c	D;E	Indicate where the study protocol can be accessed or state that a protocol was not prepared	✓
Registration	18d	D;E	Provide registration information for the study, including register name and registration number, or state that the study was not registered	✓
Data sharing	18e	D;E	Provide details of the availability of the study data	✓
Code sharing	18f	D;E	Provide details of the availability of the analytical code[4]	✓
**PATIENT & PUBLIC INVOLVEMENT**				
Patient & Public Involvement	19	D;E	Provide details of any patient and public involvement during the design, conduct, reporting, interpretation, or dissemination of the study or state no involvement.	✓
**RESULTS**				
Participants	20a	D;E	Describe the flow of participants through the study, including the number of participants with and without the outcome and, if applicable, a summary of the follow-up time. A diagram may be helpful.	✓
	20b	D;E	Report the characteristics overall and, where applicable, for each data source or setting, including the key dates, key predictors (including demographics), treatments received, sample size, number of outcome events, follow-up time, and amount of missing data. A table may be helpful. Report any differences across key demographic groups.	✓
	20c	E	For model evaluation, show a comparison with the development data of the distribution of important predictors (demographics, predictors, and outcome).	✓
Model development	21	D;E	Specify the number of participants and outcome events in each analysis (e.g., for model development, hyperparameter tuning, model evaluation)	✓
Model specification	22	D	Provide details of the full prediction model (e.g., formula, code, object, application programming interface) to allow predictions in new individuals and to enable third-party evaluation and implementation, including any restrictions to access or re-use (e.g., freely available, proprietary)[5]	✓
Model performance	23a	D;E	Report model performance estimates with confidence intervals, including for any key subgroups (e.g., sociodemographic). Consider plots to aid presentation.	✓
	23b	D;E	If examined, report results of any heterogeneity in model performance across clusters. See TRIPOD Cluster for additional details[3].	✓
Model updating	24	E	Report the results from any model updating, including the updated model and subsequent performance	✓
**DISCUSSION**				
Interpretation	25	D;E	Give an overall interpretation of the main results, including issues of fairness in the context of the objectives and previous studies	✓
Limitations	26	D;E	Discuss any limitations of the study (such as a non-representative sample, sample size, overfitting, missing data) and their effects on any biases, statistical uncertainty, and generalizability	✓
Usability of the model in the context of current care	27a	D	Describe how poor quality or unavailable input data (e.g., predictor values) should be assessed and handled when implementing the prediction model	✓
	27b	D	Specify whether users will be required to interact in the handling of the input data or use of the model, and what level of expertise is required of users	✓
	27c	D;E	Discuss any next steps for future research, with a specific view to applicability and generalizability of the model	✓

From: Collins GS, Moons KGM, Dhiman P, et al. *BMJ* 2024;385:e078378. doi:10.1136/bmj-2023-078378

[4] This relates to the analysis code, for example, any data cleaning, feature engineering, model building, evaluation.
[5] This relates to the code to implement the model to get estimates of risk for a new individual.
